# Melatonin Mitigates the Infection of *Colletotrichum gloeosporioides* via Modulation of the Chitinase Gene and Antioxidant Activity in *Capsicum annuum* L.

**DOI:** 10.3390/antiox10010007

**Published:** 2020-12-23

**Authors:** Muhammad Ali, Anthony Tumbeh Lamin-Samu, Izhar Muhammad, Mohamed Farghal, Abdul Mateen Khattak, Ibadullah Jan, Saeed ul Haq, Abid Khan, Zhen-Hui Gong, Gang Lu

**Affiliations:** 1 Department of Horticulture, College of Agriculture and Biotechnology, Zhejiang University, Hangzhou 310058, China; alinhorti@yahoo.com (M.A.); anthonylaminsamu@yahoo.com (A.T.L.-S.); mohamedfa2016@gmail.com (M.F.); 2 College of Horticulture, Northwest A&F University, Yangling 712100, China; 3 College of Agronomy, Northwest A&F University, Yangling 712100, China; izeyaar@gmail.com; 4 Department of Horticulture, The University of Agriculture, Peshawar 25120, Pakistan; mateen@aup.edu.pk (A.M.K.); saeed_ulhaq@nwsuaf.edu.cn (S.u.H.); 5 Department of Agriculture, University of Swabi, Khyber Pakhtunkhwa 9291, Pakistan; dribad@uoswabi.edu.pk; 6 Department of Horticulture, The University of Haripur, Haripur 22620, Pakistan; abidagriculturist@gmail.com

**Keywords:** anthracnose, antioxidants, biotic stress, *CaChiIII2*, chitinase, *Colletotrichum gloeosporioides*, melatonin

## Abstract

Anthracnose, caused by *Colletotrichum gloeosporioides*, is one of the most damaging pepper (*Capsicum annum* L.) disease. Melatonin induces transcription of defense-related genes that enhance resistance to pathogens and mediate physiological activities in plants. To study whether the melatonin-mediated pathogen resistance is associated with chitinase gene (*CaChiIII2*), pepper plants and Arabidopsis seeds were treated with melatonin, then *CaChiIII2* activation, hydrogen peroxide (H_2_O_2_) levels, and antioxidant enzymes activity during plant–pathogen interactions were investigated. Melatonin pretreatment uncoupled the knockdown of *CaChiIII2* and transiently activated its expression level in both control and *CaChiIII2*-silenced pepper plants and enhanced plant resistance. Suppression of *CaChiIII2* in pepper plants showed a significant decreased in the induction of defense-related genes and resistance to pathogens compared with control plants. Moreover, melatonin efficiently enabled plants to maintain intracellular H_2_O_2_ concentrations at steady-state levels and enhanced the activities of antioxidant enzymes, which possibly improved disease resistance. The activation of the chitinase gene *CaChiIII2* in transgenic Arabidopsis lines was elevated under *C. gloeosporioides* infection and exhibited resistance through decreasing H_2_O_2_ biosynthesis and maintaining H_2_O_2_ at a steady-state level. Whereas melatonin primed *CaChiIII2*-overexpressed (OE) and wild-type (WT) Arabidopsis seedlings displayed a remarkable increase in root-length compared to the unprimed WT plants. Using an array of *CaChiIII2* knockdown and OE, we found that melatonin efficiently induced *CaChiIII2* and other pathogenesis-related genes expressions, responsible for the innate immunity response of pepper against anthracnose disease.

## 1. Introduction

The genus *Colletotrichum* includes several important plant pathogens that affect several herbaceous and woody species. *Colletotrichum* is the eighth most important genus of fungi that causes plant diseases worldwide [1]. This fungus causes disease symptoms that are generally known as anthracnose in a wide range of vegetables, fruits, and other crops, including pepper [2,3,4]. In pepper, anthracnose caused by a complex of *Colletotrichum* species can cause severe yield losses at both pre- and postharvest stages during warm and rainy seasons [5]. The appearance of necrotic lesions on plant parts, including leaves, stems, flowers, and fruits, is the main symptom of this disease. Moreover, it may cause seedling blight, stem-rot and crown, etc. [6]. Many *Colletotrichum* species survive in soil or decaying organic matter and may spread through water dispersal of conidia and air transmission of ascospores from the sexual morph [7]. Among all the species, the most destructive sub-species are *Colletotrichum acutatum* and *Colletotrichum gloeosporioides* [8,9,10]. The best-known morphological characteristic of *C. acutatum* is the structure of its conidia, which have acute ends [11]. Though other conidial structures, particularly cylindrical with only one acute end, are frequently encountered, mainly in strains that have been repeatedly sub-cultured, but these conidial structures can also occur in species outside the *C. acutatum* species complex. Even the differentiation among *C. acutatum* and *C. gloeosporioides* is hard because numerous intermediate strains exist with a restricted number of typical fusiform conidia and various cylindrical ones [12]. In addition, *C. acutatum* has also been observed to form secondary conidia on the surface of living plant tissues [13].

Particularly in pepper crop, *C. gloeosporioides* is the most destructive pathogen that appears at different plant developmental stages [14]. The pathogenicity of *C. gloeosporioides* may vary on different plant parts/phases, such as vegetative, reproductive, and flowering, although, at the early stage of infection, the host cannot exhibit any apparent symptoms, but in the later period when fruiting begins, it appears on various parts of the plant [10,15]. *C. gloeosporioides* is hard to control due to its strong pathogenicity, diversity of host plant species, the susceptibility of the host plant, and the different phases of pant development, i.e., vegetative and reproductive, that it infects. Therefore, the complex growth patterns of *C. gloeosporioides* and its ability to infect a board range of host plants, and switch off the life cycle, produces difficulty in dealing with the disease it causes [16,17]. Thus far, numerous control management strategies have been employed to overcome anthracnose infection in pepper plants. These include rotation, irrigation methods, detaching the infected parts, biological control, application of different chemicals, and developing disease-resistant cultivars, etc. [18,19]. Although the development of resistant varieties is an economically and environmentally safe strategy, it is a tedious procedure that requires a long time and harder to pass through the biosafety levels [20,21,22].

The growth of pathogenic fungi is substantially controlled by particular genes of a host plant, where precise enzymes and secondary metabolites are generated by the host during the interaction with the pathogen [23]. In this regard, the transcriptomic approaches explain the regulation of pathogen-related genes during pathogens attack and their significance in gene-based biosecurity [21,22]. Therefore, it is imperative to uncover the possibility of resistance at the gene level. In this regard, the study of genes related to host interaction that controls *C. gloeosporioides* activity in plants is essential. Among all resistant genes, several chitin-binding proteins (CBP) family members, biologically functioning as pathogenic proteins (PR), increase plant innate immunity [24,25]. Typically, these genes are responsible for the degradation of the fungus cell wall through encoding lytic enzyme chitinase (EC 3.2.1.14). Chitinases have a crucial role in plant protection against diseases because they are able to digest chitin, which is the main content of many fungi cell walls [26,27]. In the pepper plant, a total of 16 members of CBP family were identified, which carry one or more chitin-binding domain(s) and natural substrate of chitin (*N*-acetyl-d-neuraminic acid), which are an integral component of binding blocks. Therefore, based on the domain organization and structural diversity, CBP genes are classified into well-conserved clades [28]. Members of the CBP family (encode chitinase enzymes) are involved in proline biosynthesis, ethylene production, and embryogenesis in plants [29]. In addition, chitinases are lytic enzymes with crucial roles in the plant defense system against fungal attacks as they have the potential to digest chitin (β-(1,4)-poly-*N*-acetyl-d-glucosamine), which is a crucial component of cell walls of many pathogenic fungi, including *C. gloeosporioides*. In addition, chitinase enzymes have an inbuilt resistance to numerous external cues [30]. Previously, the chitinase gene family has been functionally validated in several organisms, such as *Arabidopsis thaliana* [31], *Oryza sativa* [32], *Zea mays* [33], and *Capsicum annuum* [28]. However, the functional characterization in vegetable crops, especially in pepper, needs further genetic improvement to broaden our knowledge on fungal resistance strategies. Moreover, the relation between chitinase genes and *C. gloeosporioides* pathogen remains unknown. Furthermore, Virus-Induced Gene Silencing (VIGS) is considered a powerful way to measure the potential role of specific genes using a recombinant virus [34]. Here, the tobacco rattle virus (TRV)-based VIGS system was used to silence the gene in the pepper plant.

The melatonin (*N*-acetyl-5-methoxytryptamine) is an evolutionarily conserved pleiotropic molecule, having an indole ring structure, low molecular weight, and ubiquitously found in living organisms. It demonstrates pleiotropic biological activities in animals and plants [35]. In 1995, melatonin was first identified in plants [36,37]. Thereafter, melatonin has continuously drawn the attention of plant biologists due to its wide distribution and multiple roles in plants [38,39]. Being amphiphilic in character, melatonin has the ability to penetrate the cell membrane and reach mitochondria, cytosol, and even the nucleus of the cell [40]. Thus, it has a key role in non-receptor-mediated activities, including scavenging reactive nitrogen species (RNS), reactive oxygen species (ROS), improving antioxidant capacity, and oxidative stress prevention in cells, tissues, and organs [41,42,43]. ROS and RNS formation and absorption are basic processes related to physiopathology and cell biology. Therefore, it is assumed that in living organisms the primary function of melatonin is enhancement of resistance and first-line defense against biotic/abiotic stresses [44]. Plant survival, growth, and production are immensely affected by stressors, both biotic and abiotic. To combat these, plants adopt different physiological strategies. Melatonin is known to have involvement in enhancing plant physiological functions, such as normal plant development, protecting tissue injuries, and signaling environmental stress conditions [45,46,47]. Additionally, melatonin works as a growth regulator, akin to indole-acetic acid (IAA), in directing the variability of cells, tissues, and organs [48]. Previous work is primarily focused on whether melatonin is effective against abiotic stresses involving heavy metals [49], drought [50], and high salinity conditions [51]. Nevertheless, less information is available about its involvement in biotic stress mitigation, and a few questions still need answers, such as (i) Is melatonin effective against pathogen infection if applied exogenously? (ii) During the pathogen and plant interaction, does melatonin modulate ROS levels? and (iii) Is melatonin involved in promoting activities of proteins related to pathogenesis when there is plant–pathogen interaction?

In this study, we used pepper and Arabidopsis plants to determine the possible role of melatonin and chitinase (C 3.2.1.14) gene *CaChiIII2* against *C. gloeosporioides* infection through VIGS, transformation, and seed priming. Moreover, the impact of exogenous melatonin has been investigated and quantified the fluctuations in activities of several antioxidant enzymes, such as peroxidase (POD; EC 1.11.1.7), ascorbate peroxidase (APX; EC 1.11.1.11), catalase (CAT; EC 1.11.1.6), and H_2_O_2_ biosynthesis. Our focus was to determine the possible involvement and correlation of the *CaChiIII2* gene and melatonin against anthracnose disease. In this regard, a new model is presented to evaluate the stress responses mediated through melatonin. This beneficial theory may be used in further genetic improvements and is a viable way of protecting agricultural crops from pathogens.

## 2. Materials and Methods

### 2.1. Plant Material and Growing Conditions

The Pepper (*Capsicum annuum* L.) AA3 pure-line was obtained from Vegetable Plant Biotechnology and Germplasm Innovation lab, Northwest A&F University, Xianyang, China. The seeds were initially sterilized and cultured at 25 °C, 16 h/8 h light/dark for 60 days. *Arabidopsis thaliana* Columbia (Col-0) and tobacco (*Nicotiana benthamiana*) plants were grown at 22 °C under alternating 12-h light/12-h dark cycles with a photon flux density of 120 µmol m^−2^ s^−1^.

### 2.2. Antifungal Assays

An assay of antifungal activity was determined under sterile conditions using a hyphal extension-inhibition assay with a little modification [52]. A 5 mm mycelium plug of the tested fungi from an actively growing plate was inoculated into the center of a petri-dish containing potato dextrose agar (PDA) medium and different concentrations (0, 50, 100, and 500 µM) of melatonin. The plates were incubated at 28 °C for 3 days to observe the extension–inhibition of hyphae and then quantified using the ImageJ tool and photographed [53]. The major phytopathogenic fungi used in the current research included *Colletotrichum gloeosporioides* (NCBI:txid474922), *Colletotrichum acutatum* (NCBI:txid27357), and two strains of *Phytophthora capsici* (NCBI:txid4784). Three biological replicates in three individual experiments were practiced to minimize the error.

### 2.3. Inoculum Preparation

The *P. capsici* strains (PC and HX-9) and *C. gloeosporioides* pure isolation were collected from the above-mentioned lab repository. The pure spores of *C. gloeosporioides* were shacked in liquid PDA medium for 72 h at 28 °C, then the filtered suspension was centrifuged at 4000-rpm for 5 min, and the obtained spores were washed three times with deionized water. The concentration (2 × 10^5^ conidia/mL) of microspore was calculated before inoculation [54].

### 2.4. Treatments

Based on the results of the preliminary experiment (hyphal extension–inhibition assay), we selected a 100 µM concentration of melatonin for *C. gloeosporioides*. Two-month-old pepper seedlings were inoculated (sprayed) with *C. gloeosporioides* microspores described by Mello and Pessoa et al. [55,56], while the control plants were sprayed with distilled water. After inoculation, the plant leaves were sampled at different periods (0, 12, 24, 48, and 96-h post-inoculation). The pepper plants at a six-leaf stage were treated with 100 µM concentration of melatonin [57], while control plants with a mock solution and leaf samples were taken at 0, 1, 3, 6, 12, 24-h intervals. For further investigation, these samples were kept in the freezer at −80 °C. Three biological replicates in three individual experiments were practiced for validation of data. 

### 2.5. Priming Treatment

*Arabidopsis thaliana* seeds (wild-type and overexpressed lines) were hydro-primed as per the Taylor et al. [58] method using 100 µM melatonin solution and distilled water as a control. The treated seeds were re-dried at room temperature (±25 °C) for one day (time sufficient for returning to the initial water content).

### 2.6. Quantitative Real-Time PCR (qRT-PCR)

Total-RNA has been extracted from the collected samples using Trizol reagent (Invitrogen, Carlsbad, CA, USA), as described in the protocol of the manufacturers. The RNA was purified from DNA contamination with RNase-free DNase I treatment. cDNA synthesis was achieved using the Prime-Script TM RT Reagent Kit (Takara, Dalian, China). The quality and quantity of cDNA were adjusted by nanodrop, and qRT-PCR was run with gene-specific primers, as shown in Tables S1 and S2. For internal control, the ubiquitin-conjugating protein gene (*CaUbi3*) was used [59]. The relative transcript levels of all the chitinase (CaChi) genes were computed according to the 2^−ΔΔCT^ method [60].

### 2.7. Vector Construction

For the VIGS assay, the *CaChiIII2* gene was silenced using the described method by Liu et al. [61]. Precisely, the amplified target fragment 232-bp (primer pairs Table S3) of *CaChiIII2* was cloned into pTRV2 vector (Figure S1 with a set of molecular enzymes (*EcoR1*, *Xho1*). The vector cassette was transformed into an *Agrobacterium tumefaciens* strain (GV3101) as per Wang et al. method [62]. The positive clones were harvested on rifampicin (50 mg/L), gentamicin (50 mg/L), and kanamycin (50 mg/L) media. The suspension culture with OD_600_ = 1.0 was injected into the fully expanded cotyledonary leaves through a needleless syringe [63,64,65]. Meanwhile, the negative control pTRV2:00 (empty vector) and positive control pTRV2:*CaPDS* were also transformed. The plants were kept in the growth chamber, and samples were collected from *CaChiIII2*-silenced and control plants after 45 days.

The open reading frame (ORF) sequence (with no stop codon) was amplified from the full-length cDNAs of *CaChiIII2*, subsequently inserted into a pVBG2307 expression vector (Figure S2) by using the set of restriction enzymes *XbaI* and *KpnI*. The specific primer pair was synthesized by Sangon (Shanghai, China) (Table S4), and the target fragment was sequenced by Invitrogen (Shanghai, China). For overexpression analysis, a recombinant fusion vector was used to transform into Arabidopsis plants (ecotype Columbia-0, Col-0) via *Agrobacterium tumefaciens* strain GV3101 [64,66]. Putative transgenes were confirmed on 50 mmol/L kanamycin selection medium. Further homozygosity was maintained up-to T_3_ generation, and each experiment was conducted on T_3_ homozygous line.

### 2.8. Protein Localization Assay

The ORF of *CaChiIII2* was fused with green fluorescence protein (GFP) (GFP:*CaChiIII2*) (Table S5) and for transient expression transferred into pVBG-2307+GFP vector (Figure S3) driven by 35S promoter (CaMV). Tobacco epidermal cells were used for the subcellular analysis of the fused protein. The GV3101 competent cells were harvested and dissolved in 200 μM acetosyringone, 10 mM MES (pH 5.5), and 10 mM MgCl_2_, and injected into four weeks old leaves of *Nicotiana benthamiana* through a needleless syringe. The tobacco saplings were retained in the dark for two days and then in the growth chamber for three days. The *CaChiIII2* protein was visualized through a microscope (OLYMPUS, Tokyo, Japan).

### 2.9. Measurement of Antioxidant Enzymes and H_2_O_2_ Concentration 

To measure the physiological and plant defense response under *C. gloeosporioides* stress conditions, an antioxidant enzymatic assay was performed. Pepper plant material (leaf sample) 0.1 g was placed in an already chilled mortar with a small amount of quartz sand. For long term preservation, the extract was supplemented with 1% (*w*/*v*) polyvinylpolypyrrolidone, while 1.2 mL of 50 mM phosphate buffer solution (pH 7.8) containing 1 mM EDTA-Na 2 and 0.3% Triton X-100 was used to homogenize it. One micromolar ascorbate (AsA) was supplemented for ascorbate peroxidase (APX) assay in the reaction mixture. Subsequently, each homogenate extract was centrifuged at 12,000 rpm for 20 min at 4 °C, while the supernatant was used for the following assays. 

The peroxidase (POD) influence was estimated through observing absorbance increase at 470 nm owing to guaiacol oxidation (26.8 mM^−1^ cm^−1^ extinction coefficient) [67]. The function of catalase (CAT) was measured through observing absorbance decrease at 240 nm with the putrefaction of H_2_O_2_ having 39.4 mM^−1^ cm^−1^ extinction coefficient [68]. APX activity was measured through monitoring the reduction in absorbance at 290 nm as reduced ascorbate was oxidized (extinction coefficient of 2.8 mM cm^−1^) [69]. Leaf tissues (0.1 g) were extracted with 2 mL of 5% (*w*/*v*) trichloroacetic acid and centrifuged for 10 min at 12,000 rpm. An H_2_O_2_ assay was prepared with it as per Patterson et al. [70]. The data for antioxidant enzymes and H_2_O_2_ were recorded at 0, 4, 8, and 12-days post-inoculation of *C. gloeosporioides* and presented in graphs.

### 2.10. Disease Index and Histochemical Staining

Detached leaves were used for the leaf assay, as described by Ali et al. [64]. For this, *C. gloeosporioides* 5 mm small plugs were inoculated to detached leaves of the same age from pepper and placed in dark conditions for 3 days at 28 °C temperature. Disease symptoms were categorized into different levels: L_0_ = no disease symptoms, L_1_ = 1–10%, L_2_ = 11–30%, L_3_ = 31–50%, L_4_ = >50% of leaf area showing lesions. The infected area was quantified using the ImageJ tool [53], and the disease index was calculated using the formula:

$$Disease\;index=\frac{\sum \left(\mathrm{disease}\;\mathrm{level}\times \mathrm{no}.\;\mathrm{of}\;\mathrm{leaves}\;\mathrm{in}\;\mathrm{that}\;\mathrm{level}\right)\times 100}{\mathrm{Total}\;\mathrm{no}.\;\mathrm{of}\;\mathrm{leaves}\times 5}$$

Hydrogen peroxide (H_2_O_2_) was detected in the leaves of the pepper plant by DAB (3,3′-diaminobenzidine) staining. The DAB solution was injected according to Ali et al. [71]. For superoxide (^•^O_2_^−^), NBT (nitro-blue tetrazolium chloride) staining was performed according to Liu et al. [72]. Brown color spots for H_2_O_2_ and dark blue spots for ^•^O_2_^−^ were detected and filmed.

### 2.11. Statistical Analysis

Duncan’s multiple range (DMR) test was performed through SPSS 25.0 (SPSS Inc., New York, NY, USA) to evaluate the collected data statistically. The data were considered significant statistically at *p*-values ≤ 0.05. Further, the means and standard deviations (±SD) were graphed using GraphPad Prism 8.0 (GraphPad Software, Inc., LA Jolla, CA, USA).

## 3. Results

### 3.1. Effect of Melatonin on Oomycete and Fungi Growth

**Figure 1:**
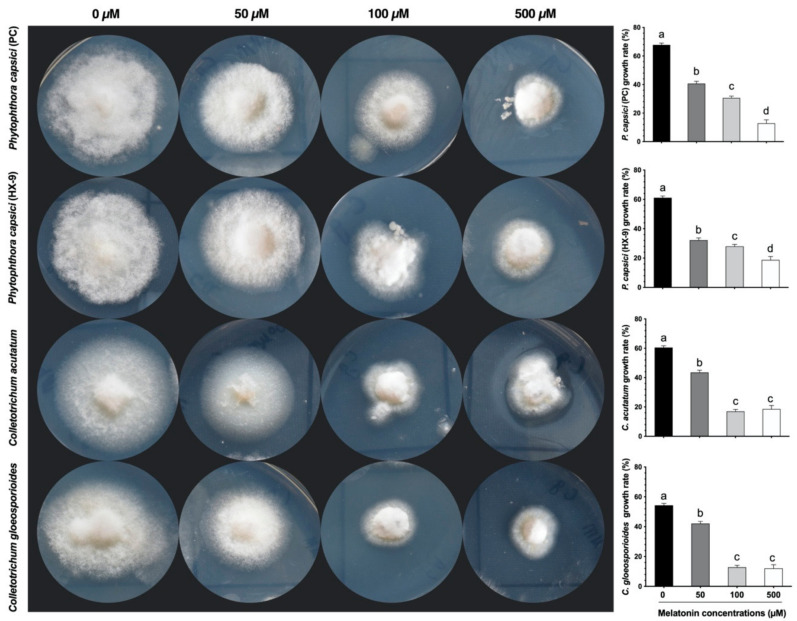
In-vitro apyrase inhibition effects of melatonin on the growth of different fungi and oomycetes. Representative photographs of assay plates of *Colletotrichum gloeosporioides*, *Colletotrichum acutatum*, and two strains of *Phytophthora capsici* (PC and HX-9) in the presence of melatonin concentrations: 0, 50, 100, and 500-µM, cultured on potato dextrose agar (PDA) medium. Here the mean values ± SDs of three replications are given. Lower case letters (a–d) on each histogram show differences at *p* ≤ 0.05 using the Duncan Multiple Range (DMR) test.

In the in-vitro study, we examined the antifungal effect of exogenous application of melatonin at concentrations of 0, 50, 100, and 500 µM. As shown in Figure 1, melatonin showed broad-spectrum activity and inhibited the growth/extension of tested phytopathogenic fungi: *Colletotrichum gloeosporioides*, *Colletotrichum acutatum*, and two strains of *Phytophthora capsici* (PC and HX-9). After 5 days, the growth rate of all tested phytopathogenic fungi was normal in control (0 µM) plates. At 50 µM concentration, the antifungal activity of melatonin was not obvious on *P. capsici* strains compared to *Colletotrichum* species. A slight inhibition in the hyphal growth of *P. capsici* strains (PC and HX-9) was noted at 100 and 500 µM. Compared with control (no-melatonin), 100 µM melatonin dose exhibited the highest decline (>76%) in the hyphal extension of *C. gloeosporioides*, closely followed by a >71% decrease in *C. acutatum* at the same concentration. Thus, 100 µM melatonin treatment remarkably reduced *C. gloeosporioides* hyphal extension, provided maximum efficiency against fungal growth (Figure 1). 

### 3.2. Expression Pattern of Chitinase Genes

**Figure 2:**
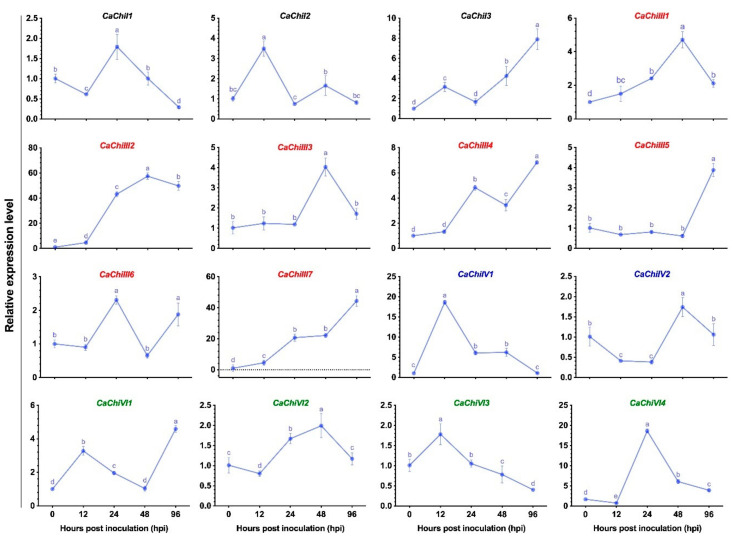
The transcript levels of chitin-binding protein family genes in response to *C. gloeosporioides* infection. Tissue samples were collected at different intervals (0, 12, 24, 48, and 96-h post-inoculation) and were examined by qRT-PCR. The colors represent classes: black: I, red: III, blue: IV, and green: VI. Here the mean values ± SDs of three replications are given. Lower case letters (a–e) on each line show significant differences at *p* ≤ 0.05 using the Duncan Multiple Range (DMR) test.

The expression pattern of pepper CBP encoding genes was analyzed under *C. gloeosporioides* post-infection, based on transcriptomic data [28]. The obtained results showed a differential expression pattern of pepper chitinase genes. All 16 genes were upregulated, and no gene was completely downregulated at all tested points (Figure 2). Initially, at 12 h post-inoculation (hpi) treatment, most of the genes did not show any significant infection response, while >36% of the genes were downregulated, and then expression increased abruptly at 24-hpi. However, 31% of genes (*CaChiI1*, *CaChiIII1*, *CaChiIII2*, *CaChiIII4*, and *CaChiVI7*) progressively showed increased transcript levels at each point and reached a peak. Nevertheless, two genes (*CaChiI1* and *CaChiIV2*) showed no substantial transcript level changes against *C. gloeosporioides* infection and mostly were not upregulated at different intervals. Compared to other chitinase genes, *CaChiIII2* and *CaChiIII7* exhibited remarkable expression levels, i.e., >57-fold at 48-dpi and >44-fold at 96-dpi, respectively. Thus, they were considered as candidate genes for further investigation of plant resistance against *C. gloeosporioides* infection. Pepper chitinase class-III genes showed a higher response to *C. gloeosporioides* than the members of other classes, such as I, IV, and VI (Figure 2). Generally, we found that the CBP gene family is substantially influenced by *C. gloeosporioides* during a period of inoculation.

**Figure 3:**
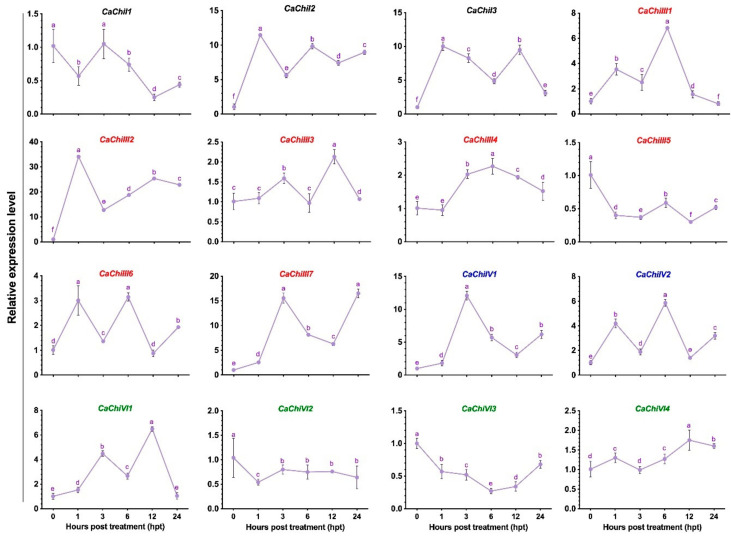
Effect of exogenous melatonin application on the transcription of chitin-binding protein family genes. Tissue samples were taken at different intervals (0, 1, 3, 6, 12, and 24-h post-treatment) and were analyzed through qRT-PCR. The different colors show classes: black: I, red: III, blue: IV, and green: VI. The mean values ± SDs of three replications are given. Lower case letters (a–f) on each line show significant differences at *p* ≤ 0.05 using the DMR test.

As expected, exogenous melatonin application induced 66 differentially expressed genes (DEGs) for different plant defense mechanisms, such as stress tolerance, fungicide resistance, and virulence [73]. To understand the response of specific defense-related genes, the expression levels of pepper chitinase genes response to 100 µM melatonin treatment was analyzed by qRT-PCR. The transcript level of 75% of the genes (*CaChiI2*, *CaChiI3*, *CaChiIII1*, *CaChiIII2*, *CaChiIII3*, *CaChiIII4*, *CaChiIII6*, *CaChiIII7*, *CaChiIV1*, *CaChiIV2*, *CaChiVI1*, and *CaChiVI4*) was upregulated during the whole treatment period, while 25% of the genes (*CaChiI1*, *CaChiIII5*, *CaChiVI2*, and *CaChiVI3*) showed a downregulated trend (Figure 3). Among all the members, *CaChiIII2* was induced more remarkably during the whole treatment period, displaying the highest expression level compared with other genes, and the transcript peaked (34-folds) at 1-h post-treatment (hpt). Based on the response to exogenous melatonin treatment, class-III members showed significant response followed by class-I, while class-VI members exhibited either the lowest expression or downregulation (in most cases).

Furthermore, the expressions of other genes involved in the defense system of pepper were also investigated (Figure S4). Under melatonin treatment, transcript levels of several genes were significant, while relatively higher expressions were detected for *CaPO2*, *CaASMT1*, *CaSNAT1*, *2*, *CaICS1*, *CaMPK3*, *6*, and *CaCAT1* at all tested time points. *CaDEF1* and CaAPX1 showed similar expression patterns, which were upregulated and reached the highest peak at 24 (3.5-fold) and 12-hpt (3-fold), respectively. Initially, most of the genes showed an abrupt response to melatonin treatment, and expression remained elevated compared with 0-hpt. However, decreased expression was also observed for some genes at certain time points.

### 3.3. Protein Localization Assay 

**Figure 4:**
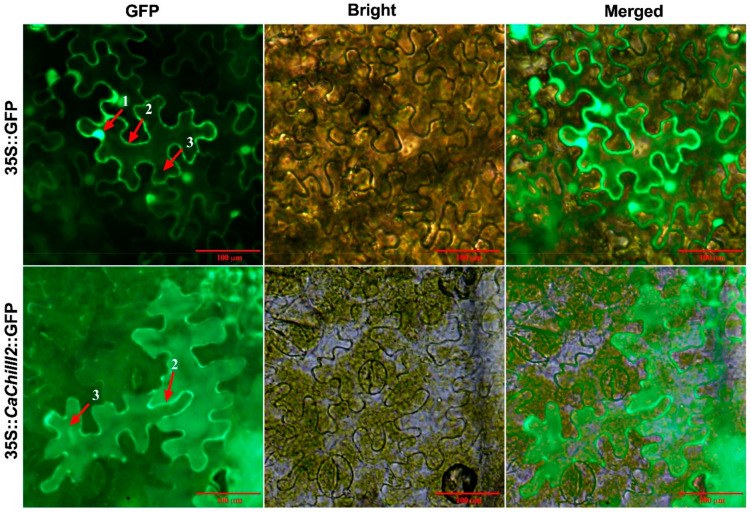
Representative images of protein localization assay of pepper chitinase gene *CaChiIII2* in tobacco leaves epidermal cells. 35S∷ green fluorescence protein (GFP) represents control. A bright fluorescent field was used to measure the fluorescence. The numbers 1, 2, and 3 represent the nucleus, cell membrane, and cytoplasm, respectively.

The pVBG2307 expression vector containing 35S promoter and green fluorescence protein (GFP) reporter gene was recombined with the ORF portion of *CaChiIII2*. The *Nicotiana benthamiana* plants were infiltrated with pVBG2307∷GFP, and pVBG2307∷*CaChiIII2*∷GFP fused plasmids for *CaChiIII2* expression in ephemeral tissue [74]. The confocal laser micrographs exhibited that 35S∷*CaChiIII2*∷GFP fused protein was mostly localized in the cell membrane and cytoplasm of the cell (Figure 4). Moreover, the mock pVBG2307∷GFP vector (control) gave signals in the membrane, cytoplasm, and nucleus of the cell.

### 3.4. CaChiIII2 Knockdown in Pepper Confers Susceptibility to C. gloeosporioides

**Figure 5:**
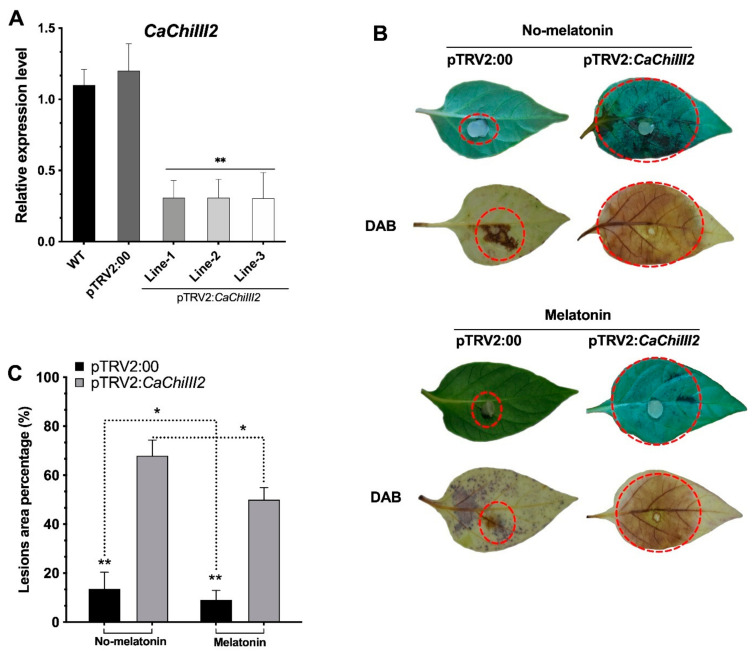
Silencing efficiency of *CaChiIII2* and lesions area of detached leaves after the inoculation of *C. gloeosporioides*. (**A**) The relative expression level of *CaChiIII2* in wild-type (WT), TRV2:00 and *CaChiIII2*-silenced lines of pepper plants, (**B**) *C. gloeosporioides* infection symptoms appeared on leaves of TRV2:*CaChiIII2* and TRV2:00 plant, H_2_O_2_ detection with DAB (3,3-diaminobenzidine) staining, (**C**) Percent infection of *C. gloeosporioides* on pepper leaves in both groups (melatonin treated and untreated). The bars denote mean values ± SD of three individual replications, while asterisks on the bars denote differences among means at *p* ≤ 0.05 (*) and *p* ≤ 0.01 (**) according to the DMR test.

To investigate the impact of loss-of-function of the target gene in pepper cultivar AA3, the virus-induced gene silencing (VIGS) technique has was for the knockdown. To determine the effectiveness of VIGS, we first examined the ability of TRV2 by silencing the marker gene pepper phytoene desaturase (*CaPDS*). We obtained a fragment of the pepper PDS gene, 302-bp (Table S3), and used the conventional syringe infiltration procedure to induce VIGS in the initial experiments. TRV2:*CaPDS* was able to induce a photobleaching phenotype that occurred in the absence of the gene product. Ten days after infiltration, the photobleaching phenotype of PDS was evident in the upper leaves of treated plants (Figure S5). After 10 days, the phenotype was seen in newly expanding leaves, and the effect persisted permanently. Expression analysis exhibited a devastating decrease in *CaPDS* expression in TRV2:*CaPDS* plants. As a negative control, the TRV2:00 vector was used. Simultaneously, the silencing proficiency of pepper chitinase gene *CaChiIII2* was examined in different lines by qRT-PCR analysis. The result showed that the expression level of the target gene in pTRV2:*CaChiIII2* (*CaChiIII2*-silenced) plants were substantially lower (Line1 > 76%, Line2 > 75%, and Line3 = 78%) than that of pTRV:00 plants (Figure 5A). 

Furthermore, the function of the *CaChiIII2* gene and the effect of melatonin on the pepper plant resistance response was established through a detached leaf assay. Three days after inoculation, *C. gloeosporioides* injuries were observed on the leaf dorsal side of both TRV2:*CaChiIII2* and TRV2:00 plants of the no-melatonin pretreated group. However, the infected area of the TRV2:*CaChiIII2* was substantially larger (>83%) than those of the TRV2:00 plants. In the melatonin pretreated group, the infected leaf area was 50% in TRV2:*CaChiIII2* plant, which was 82% higher than TRV2:00 (9%) (Figure 5B,C). The quantitative analysis revealed that due to the knockdown of *CaChiIII2*, the disease-infected area increased. Whereas the exogenous melatonin application restricted the growth of *C. gloeosporioides* infection to a larger extent, i.e., a 26% reduction in pTRV2:*CaChiIII2* and 33% in pTRV2:00 plants. Generally, the generation of reactive oxygen species (ROS) is a common plant response under stress conditions [75]. The hydrogen peroxide (H_2_O_2_) biosynthesis was observed in pTRV2:*CaChiIII2* plant leaves and turned brownish when stained with 3,3-diaminobenzidine (DAB). The development of brownish color on leaves with DAB staining indicated H_2_O_2_ accumulation (Figure 5B).

### 3.5. Melatonin Impacts on CaChiIII2 Silencing

**Figure 6:**
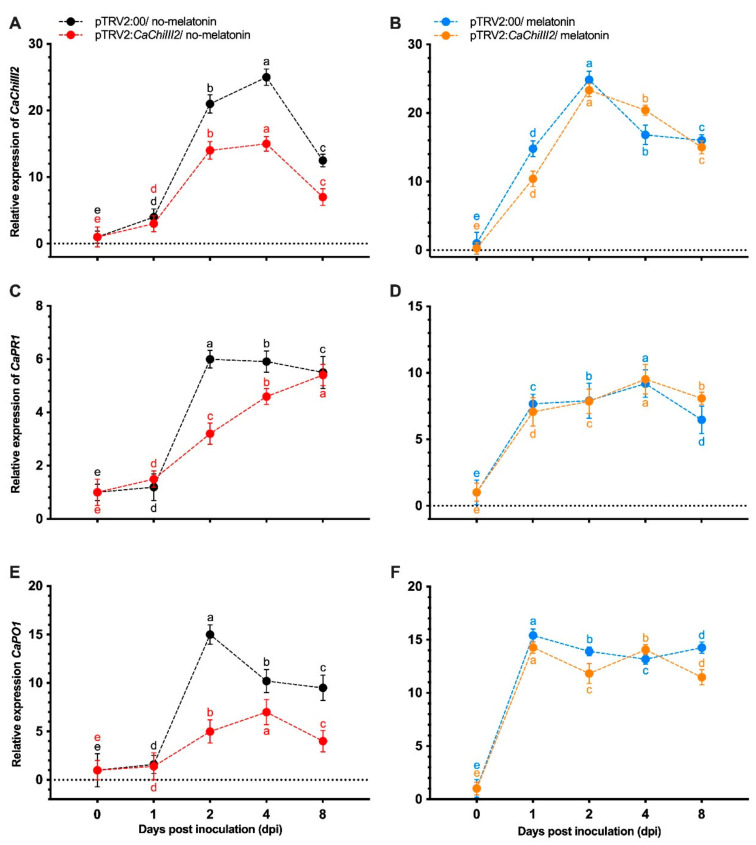
Effect of *CaChiIII2* knockdown and exogenous melatonin pretreatment on the defense response of pepper plant against *C. gloeosporioides*. (**A**) The transcript level of *CaChiIII2* without melatonin pretreatment in pTRV2:00 and pTRV2:*CaChiIII2,* (**B**) 100 μM melatonin pretreated, (**C**) Transcript level of *CaPR1* without melatonin pretreatment in pTRV2:00 and pTRV2:*CaChiIII2*, (**D**) 100 μM melatonin pretreated, (**E**) Transcript level of *CaPO1* without melatonin pretreatment in pTRV2:00 and pTRV2:*CaChiIII2*, (**F**) 100 μM melatonin pretreated. The mean values ± SDs of three replications are given. Lower case letters (a–f) on each line show the respective differences at *p* ≤ 0.05 using the DMR test.

After the successful knockdown of our target gene *CaChiIII2*, the transcript level under *C. gloeosporioides* significantly decreased, i.e., >33 and 40% at 2 and 4-dpi, respectively, in pTRV2:*CaChiIII2* (*CaChiIII2*-silenced) as compared to pTRV2:00 (control) plants at all tested time points. On the other hand, melatonin treatment increased the expression of the *CaChiIII2* gene in both sub-groups (pTRV2:00 and pTRV2:*CaChiIII2*) compared to the no-melatonin group (Figure 6A,B). The application of exogenous melatonin (100 µM) recovered the expression of the silenced gene, which might be due to triggering the salicylic acid (SA) pathway because we previously showed that the *CaChiIII2* gene positively responded to exogenous SA treatment [28]. 

To confirm whether *CaChiIII2* knockdown had any transcription altering effect, the levels of expression of other genes involved in the defense mechanism were also investigated. It was observed that the expression levels of *CaPR1* [76] and *CaPO1* increased in response to *C. gloeosporioides* infection. However, in pTRV2:*CaChiIII2* the transcript levels of both genes were substantially lower than pTRV2:00 plants in the no-melatonin group (Figure 6C,E). On the other side, in the melatonin pretreated group, the expression of both defense-related genes was dramatically elevated in pTRV2:*CaChiIII2* plants and reached the highest peak as TRV2:00 during *C. gloeosporioides* interaction (Figure 6D,F). On 8-dpi, the transcription of *CaPR1* was 25% higher in pTRV2:*CaChiIII2* plants relative to pTRV2:00 within the 100 µM melatonin treated group. In the no-melatonin group, there was a remarkable difference in the expression level of the same gene in *CaChiIII2*-silenced and control plants. So, melatonin treatment recovered the loss of the function of *CaChiIII2* and also other associated genes, which exhibited high expression that was maintained during the rest of the infection period (Figure 6).

### 3.6. Antioxidant Enzymes and Peroxidase 

**Figure 7:**
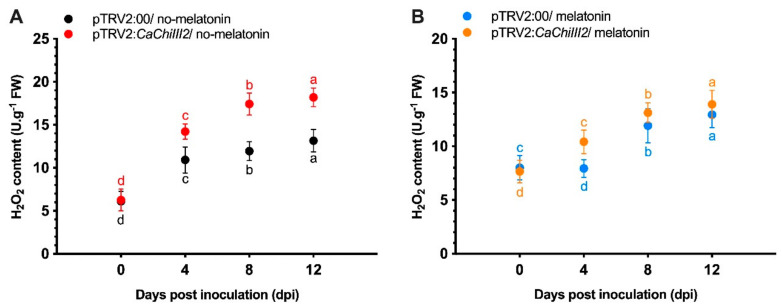
Effect of *CaChiIII2* knockdown and exogenous melatonin application on H_2_O_2_ content in pepper leaves inoculated with *C. gloeosporioides*. (**A**) Pepper leaves pretreated with a mock solution, (**B**) 100 μM melatonin pretreated. Mean values ± SDs for five replicates are presented. Lower case letters (a–d) represent significant differences at *p* ≤ 0.05 using the DMR test.

Compared with control (pTRV:00), the *CaChiIII2*-silenced (pTRV2:*CaChiIII2*) pepper plants had significantly higher H_2_O_2_ contents at 8 and 12-dpi in the no-melatonin group (Figure 7A). Whereas in both cases, melatonin treated and untreated, the *CaChiIII2*-silenced (pTRV2:*CaChiIII2*) plants had a highly significant concentration of H_2_O_2_ contents relative to control (pTRV2:00). With melatonin post-treatment, the contents remarkably decreased but obviously in pTRV2:*CaChiIII2* at 8 and 12-dpi (Figure 7B). 

**Figure 8:**
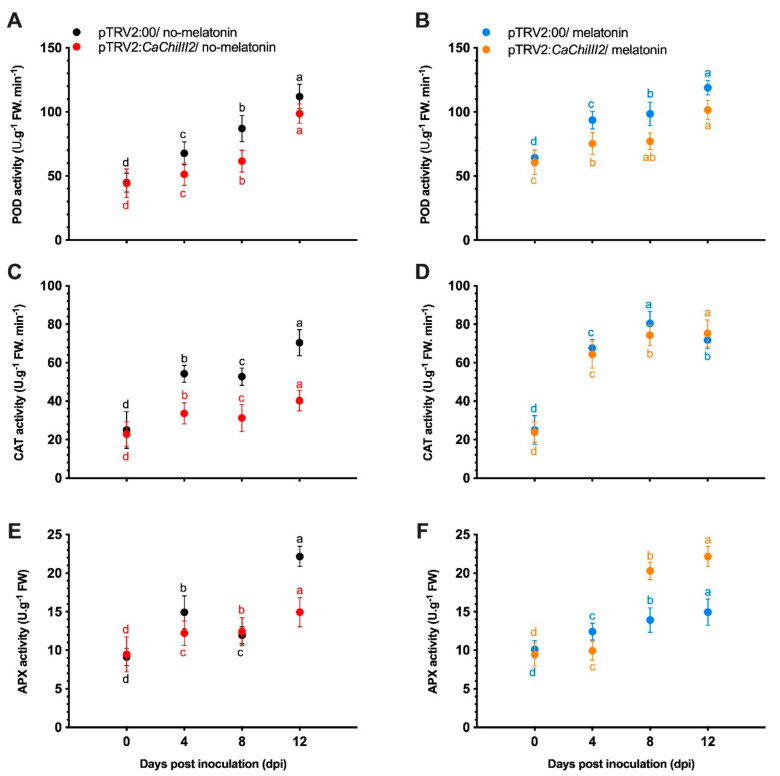
Effect of *CaChiIII2* knockdown and exogenous melatonin treatment on antioxidant enzyme activities during *C. gloeosporioides* infection in pepper. (**A**) POD activities without melatonin pretreatment in pTRV2:00 and pTRV2:*CaChiIII2*, (**B**) 100 μM melatonin pretreated, (**C**) CAT activities without melatonin pretreatment in pTRV2:00 and pTRV2:*CaChiIII2*, (**D**) 100 μM melatonin pretreated, (**E**) APX activities without melatonin pretreatment in pTRV2:00 and pTRV2:*CaChiIII2*, (**F**) 100 μM melatonin pretreated. Mean values ± SDs of five replications are provided. Lower case letters (a–d) respective differences at *p* ≤ 0.05 with the DMR test.

Activities of antioxidant enzymes involved in scavenging of H_2_O_2_ during the pathogen infection periods are shown in Figure 8. After the inoculation of *C. gloeosporioides*, the antioxidant enzymes (POD, CAT, and APX) decreased with the prolongation of infection time in both *CaChiIII2*-silenced (pTRV2:*CaChiIII2*) and control (pTRV2:00) plants. However, the decline in pTRV2:*CaChiIII2* was more severe than pTRV2:00 plants, which might have increased the pepper plant susceptibility. In the no-melatonin group, the activity of POD was lower, while in the melatonin pretreatment group, the activities substantially increased in both sub-groups (pTRV2:00 and pTRV2:*CaChiIII2*) during the interaction with *C. gloeosporioides* (Figure 8A,B). Especially, this was more obvious in the initial stage of exposure to a pathogen, showing that melatonin pretreatment at 100 µM concentration boosted POD activity. This enhancement in the POD function remained effective during the whole stress phase. In the no-melatonin group, the CAT activity in *CaChiIII2*-silenced plants was 37, >40, and 57% lower than pTRV2:00 at 4, 8, and 12-dpi, respectively (Figure 8C). However, 100 µM melatonin pretreatment distinctively increased the overall CAT activity in both sub-groups (pTRV2:00 and pTRV2:*CaChiIII2*) and also decreased the deviation up to 5, 7.6, and −4% at 4, 8, and 12-dpi, respectively (Figure 8D). Initially, the APX activity abruptly increased in the no-melatonin group on 4-dpi. Then the highest activity (22.16-fold) was noted at 12-dpi in pTRV2:00, which was >32% higher than pTRV2:*CaChiIII2*. On the other hand, the melatonin treated group did not display a remarkable response at 4-dpi, whereas the APX activity increased only in pTRV2:*CaChiIII2* plants at 8 and 12-dpi (Figure 8E,F). These results reveal that melatonin interaction was more with CAT than with POD and APX

### 3.7. Overexpression of CaChiIII2 and Melatonin Application

**Figure 9:**
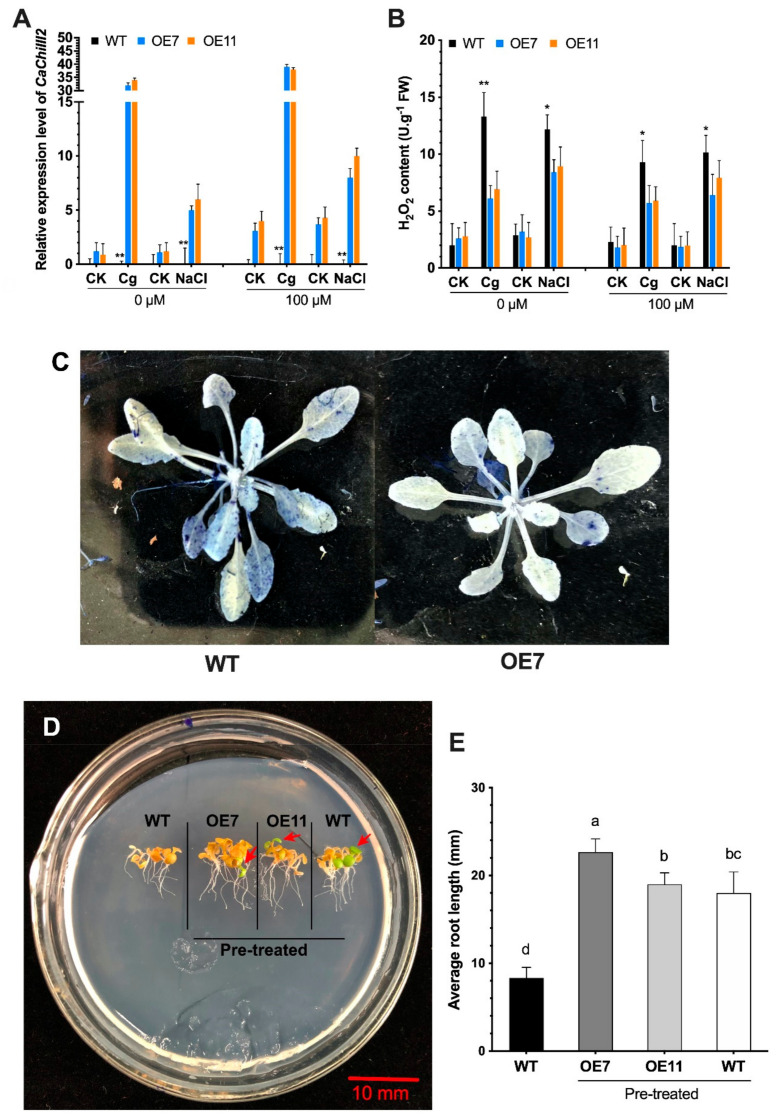
The effect of *CaChiIII2*-overexpression and melatonin treatment on Arabidopsis stress tolerance. (**A**) The expression level of *CaChiIII2* in Cg (*C. gloeosporioides*) infected, NaCl treated and CK (control) pants, one group was pretreated with 0 µM and another with 100 µM melatonin, (**B**) H_2_O_2_ concentrations in WT and transgenic lines, (**C**) Nitro-blue tetrazolium chloride (NBT) staining showing ^•^O_2_^−^ concentrations in WT and *CaChiIII2*-overexpressed lines, (**D**) The phenotypes showed root length of unprimed (WT) and melatonin primed Arabidopsis WT and transgenic lines growing on MS medium supplemented with 300 mM/L NaCl, (**E**) Graphical presentation of root length (mm). Mean values ± SDs of five replications are provided. Asterisks and lower-case letters (a–d) on the bars denote differences among means at *p* ≤ 0.05 (*) and *p* ≤ 0.01 (**) according to the DMR test.

The most *C. gloeosporioides* resistant homozygous T_3_ Arabidopsis lines, i.e., *CaChiIII2*-overexpressed line-7 and 11 (OE7 and OE11), and wild-type (WT) were selected for further analysis. One group (melatonin pretreated) of *CaChiIII2-*overexpressed lines and WT plants were inoculated with *C. gloeosporioides*, and the other group was investigated for NaCl stress (300 mM) tolerance. The results revealed that melatonin (100 µM) pretreated OE7 and OE11 lines provided the highest response relative to WT plants and the non-treated (0 µM) group under both stresses (*C. gloeosporioides* and NaCl). Whereas the response of *CaChiIII2*-overexpressed plants under biotic stress was higher than those under abiotic stress, i.e., 79% (OE7) and 74% (OE11) (Figure 9A). Furthermore, the accumulation of H_2_O_2_ in *CaChiIII2* transgenic lines (melatonin pretreated group) was remarkably lower than WT after the *C. gloeosporioides* infection and NaCl stress (Figure 9B). Whereas, we noted the lowest concentration of H_2_O_2_ content in OE7 and OE11 (melatonin 100 µM pretreated group) during *C. gloeosporioides* infection relative to NaCl treated plants, which suggest a particular role of *CaChiIII2* against fungus (degradation of the fungus cell wall). Additionally, a few dark blue spots were noticed, mostly on the leaves of *CaChiIII2*-overexpressed Arabidopsis plants when treated with nitro-blue tetrazolium chloride solution, which suggests that the biosynthesis of superoxide (^•^O_2_^−^) is lower in transgenic seedlings compared with WT ones (Figure 9C).

In addition, melatonin primed seeds of Arabidopsis transgenic lines (OE7 and OE11) and WT were grown on MS media to investigate NaCl stress tolerance by measuring root length. The WT unprimed seeds were grown on the same media as a control. As shown in Figure 9D,E, the roots of the *CaChiIII2*-OE line were considerably longer than those of the WT (unprimed) seedlings. When WT seeds were primed with the same melatonin concentration, they also exhibited an increase in root length and closely followed the length of the OE lines. Whereas, in comparison with unprimed WT seedlings, melatonin priming significantly increased the root length of the WT plant. Additionally, seeds primed with 100 µM melatonin noticeably decreased the mortality rate of OE and WT seedlings relative to unprimed WT under stress conditions (Figure 9D). This indicates that melatonin triggers the defense-related genes, including *CaChiIII2* to increase the defense response of a plant to encounter stress.

## 4. Discussion

To combat different microbial pathogens and ecological issues, plants develop various processes to oppose cell and plant damage due to these biotic and abiotic stresses [39,77,78]. Our results demonstrated that pepper chitinase gene (*CaChiIII2*) and melatonin application had a protective function against *C. gloeosporioides* infection in pepper plants. It was obvious due to the reduction in the lesioned areas and their expansion rate. Moreover, pretreatment of melatonin helped increase the potential efficiency of *CaChiIII2* and other defense-related genes (*PR1* and *PO1*) compared with that of pTRV2:*CaChiIII2*/no-melatonin plants. Consistently, it was observed that the 100 µM melatonin level was the best for protection from *C. gloeosporioides* infection (Figure 1). A similar concentration was effective in plant resistance to virus infections [57]. In Arabidopsis seedlings, it also enhances lateral bud development and the growth of adventitious roots [79]. However, it was first observed here that the chitinase gene and exogenous application of melatonin had a mitigating effect against *C. gloeosporioides* infection in pepper. 

This work was established based on some crucial concerns. We observed that the chitinase gene *CaChiIII2* improved resistance against *C. gloeosporioides* in pepper and Arabidopsis (Figure 5 and Figure 9). This is a new finding which shows the role of the chitin-binding protein family member *CaChiIII2* in plant inherent resistance to *C. gloeosporioides* pathogen. Additionally, the *CaChiIII2* transcripts enhanced considerably and rapidly when treated with 100 µM melatonin as well as *C. gloeosporioides* inoculation (Figure 2 and Figure 3). Together with the induction of exogenous melatonin + *C. gloeosporioides* inoculation (Figure 6B), these results indicated the positive role of melatonin and *CaChiIII2* during a stress response. Moreover, the transcript of other defense-related genes, such as *CaPRI* and *CaPO1,* were rapidly and highly induced in melatonin pretreated pepper plants (pTRV2:*CaChiIII2*/melatonin) compared with the mock treatment (pTRV2:*CaChiIII2*/no-melatonin) (Figure 6). *CaChiIII2* and *CaPR1* transcript levels are the most abundant and are highly induced by salicylic acid (SA) and pathogens [28,80,81]. Thus, the PR genes are generally used as typical markers for the SA-mediated plants′ inherent resistant response [82,83]. Further, the treatment of exogenous melatonin also induced the *CaChiIII2* gene after a short lag time of 1 h. This suggests the action of melatonin to be an SA akin signaling molecule (Figure 3). Additionally, the application of exogenous melatonin treatment significantly enhanced the transcript levels and melatonin biosynthesis genes (Figure S4). So far, it has been observed that melatonin-elicited pathogen had shown specificity to an avirulent pathogen and are associated with the resistance (R) gene signaling pathway [84]. Moreover, high evolutionary conservation of SNAT (serotonin N- acetyltransferase) genes over a long period of time in various organisms suggests that SNAT plays an important function in the production of melatonin. The most prominent role of melatonin is its participation in the plant defense mechanism under various stresses [85,86]. Additionally, melatonin may act as a crucial molecule in the development and survival of plants. Whereas, some recent research noted that melatonin is closely associated with the innate immune response in plant–pathogen interactions [87,88]. The application of exogenous melatonin enhances plant resistance to bacterial and fungal infection. There is an obvious correlation between plant resistance against pathogen and melatonin, which is well supported by the inducement of expression of plant defense genes. Though, some previous observations indicated that melatonin-elicited pathogen resistance to *Pseudomonas syringe* in Arabidopsis followed SA-dependent manner [88].

As a PR gene, *CaChiIII2* and other defense relative genes, including *CaPR1* and *CaPO1,* protection of pepper plants from *C. gloeosporioides* contamination can be dualistic. It has been formerly demonstrated that they can work directly on the fungi through weakening and deteriorating their cell walls [89,90]. Second, oligosaccharide elicitors, released through digested walls, can induce a consequent chain of defense reactions [91]. Chitinase activities can be substantially induced by infection, as reported in several pathosystems [92]. For instance, in the event of a *Fusarium solani* infestation in pea pods, the activity of chitinase genes is enhanced up to 9-fold [93]. It has also been detected that in melatonin pretreated plants, the transcript induction and activity of chitinase gene *CaChiIII2* substantially increased at the beginning of inoculation and lingered on until the end of the experiment. This proves that melatonin has the ability to enhance and sustain the *CaChiIII2* transcript level and thus can contribute towards greater protection of pepper plants against fungal infections. In addition, to inducing plant innate immunity against pathogen infection, exogenous application of melatonin had a direct effect and substantially attenuated the infection of *C. gloeosporioides, C. acutatum,* and *P. capsici* (PC and HX-9) by inhibiting hyphal growth (Figure 1). For the first time, this study disclosed the direct inhibitory effects of melatonin on the growth of *C. gloeosporioides*. The observations provide new understandings of the processes involved in the direct plant pathogens and melatonin interaction.

H_2_O_2_ is regarded as one of the most useful and steady reactive oxygen species (ROS), with numerous promising purposes in plants’ protection strategies [94]. Further to its direct antimicrobial effects, such as inhibiting the germination of the spores of various fungal pathogens, H_2_O_2_ is involved in the oxidative cross-linking of cell wall glycoproteins [95]. Under stress conditions, the increase in ROS generally damages the cell membrane, causing cell death [96]. Additionally, previous studies indicate that exogenously applied melatonin significantly suppress the biosynthesis of hydroxyl radicals and H_2_O_2_ [51]. Further, Chen et al. [97] proposed that external melatonin treatment lessened ROS production caused by salt stress in maize leaves. In the present study, inoculation with *C. gloeosporioides* was associated with a substantial increase in H_2_O_2_ content at 4, 8, and 12-dpi in pTRV2:*CaChiIII2*/no-melatonin. However, a remarkable decrease was noted in *CaChiIII2*-silenced pepper plants after melatonin treatment (Figure 7B). When we compared pTRV2:*CaChiIII2*/no-melatonin plants with pTRV2:00 and pTRV2:*CaChiIII2* of the melatonin treated group, *CaChiIII2* and melatonin relatively inhibited the excessive biosynthesis of ROS. Similarly, melatonin treatment is correlated with reduced ROS accumulation under abiotic stresses, such as chilling or high salinity stress [51,98]. This is maybe because of a link with the amounts of ROS accumulated. Therefore, during biotic or abiotic stresses, melatonin could restrain ROS amounts by keeping intracellular H_2_O_2_ concentrations stable and reducing stress-triggered ROS accumulation. This, in turn, prevents plant tissues from oxidative damage.

APX, POD, and CAT, are essential scavenging enzymes, which eliminate H_2_O_2_ through the Halliwell–Asada–Foyer pathway mechanism [99]. When we inoculated plants with *C. gloeosporioides*, the POD, CAT, and APX levels significantly decreased in pTRV2:*CaChiIII2*/no-melatonin at 4, 8, and 12-dpi compared with those of pTRV2:00/no-melatonin (Figure 8). It signifies that *CaChiIII2*-knockdown makes the pepper plant more prone to pathogenicity. Moreover, the new findings of this study were that melatonin pretreatment (pTRV2:*CaChiIII2*/melatonin) had a remarkable effect on CAT activity when compared with pTRV2:*CaChiIII2*/no-melatonin plants, indicating a link between CAT activity and 100 µM melatonin treatment (Figure 8D). The CAT activity increased in melatonin pretreated plants and persisted over the experimental period. These results are consistent with the pattern found for H_2_O_2_ accumulation, which indicates that pathogen infection has no effect on the system of oxidation–reduction of melatonin treated plants. Similarly, it also proposes that melatonin has a considerable function to keep the system of oxidation–reduction at its optimum. A complex protein family comes from peroxidases. These proteins have a role in catalyzing various substrates oxido-reduction through H_2_O_2_ [100]. Peroxidases are involved in wall-building processes, e.g., oxidation of phenols, suberization, and lignification of host cells during the defense reaction against pathogenic agents [101]. Therefore, these are essential mechanisms related to the response of plants against pathogen attacks. Further, this study showed that POD and APX activities were significantly elevated during the *C. gloeosporioides* inoculation period in pTRV2:00/no-melatonin plants. However, the activity was weaker in *CaChiIII2*-silenced (melatonin pretreated and no-melatonin) plants. It demonstrated that both the enzymes (POD and APX) activity and the effect of exposure to 100 µM melatonin was not considerably large as compared to CAT activity. These findings are also confirmed by the expression analysis, which exhibited that the response of *CaCAT1* was stronger and highly significant relative to *CaAPX1* gene after melatonin treatment (Figure S4). These changes could increase the cell walls’ efficiency to become barriers that slow down the dispersal of a disease. Moreover, enhanced POD activity is considered to improve resistance against pathogens in rice [102] and wheat [101]. 

In the in-vivo study, the transgenic Arabidopsis ectopically expressed *CaChiIII2* showed higher expression levels relative to the melatonin untreated group (0 µM melatonin) under *C. gloeosporioides* infection and NaCl stress (Figure 9A). More importantly, the transcript of *CaChiIII2* in pepper leaves was highly induced by melatonin treatment compared to other chitinase genes, suggesting the possible interaction in response to melatonin (Figure 3). On the other side, pre-sowing treatment (seed priming) with melatonin significantly increased the root-length of transgenic Arabidopsis plants under NaCl stress. Moreover, it should be noted that root length is substantially longer in melatonin primed seeds of *CaChiIII2*-overexpressed lines (OE7 and OE11) as compared to melatonin primed WT plants and non-primed WT (Figure 9D). Previous studies suggested that melatonin application enhanced root length in melon and rice seeds treated with melatonin improves seeds germination and growth after germination during both normal and stressful circumstances [103,104]. The roots elongation and regeneration strongly depend on the indole-acetic acid (IAA) amount and its movement to the roots generation zone (adventitious or lateral promotion) [105]. Whereas melatonin is structurally related to IAA, and both are indole derivatives [106]. Such a positive effect of exogenous melatonin application and overexpression of *CaChiIII2* on root-length is consistent with the hypothesis that melatonin may exhibit some auxin-like effects in plants [48,107]. These findings corroborate that melatonin is implicated in the regulation of the *CaChiIII2* gene in promoting the growth and development of Arabidopsis seedlings and improves plant defense mechanisms. 

Melatonin pretreated (seed priming) WT and transgenic Arabidopsis plants ectopically expressing *CaChiIII2* showed obvious resistance to NaCl stress by reduced mortality and chlorophyll degradation (Figure 9D). One of the underlying mechanisms may be that the melatonin pretreatment triggers *CaChiIII2* and other defense-related genes by activating the SA pathway, and thus plants show resistance [88]. The cold stress defensive action of melatonin has been revealed by the up-regulation of some cold-signaling gene expression, including CBFs and antioxidant genes (*ZAT10* and *ZAT12*) that are the main ROS transcription activators [108]. Moreover, melatonin plays an important role in enhancing antioxidant activities of plants growing under abiotic stress conditions [109,110,111]. In various crops, exogenously applied melatonin is reported to improve photosynthetic activity and augment chlorophyll formation in a stressful environment [112,113,114].

**Figure 10:**
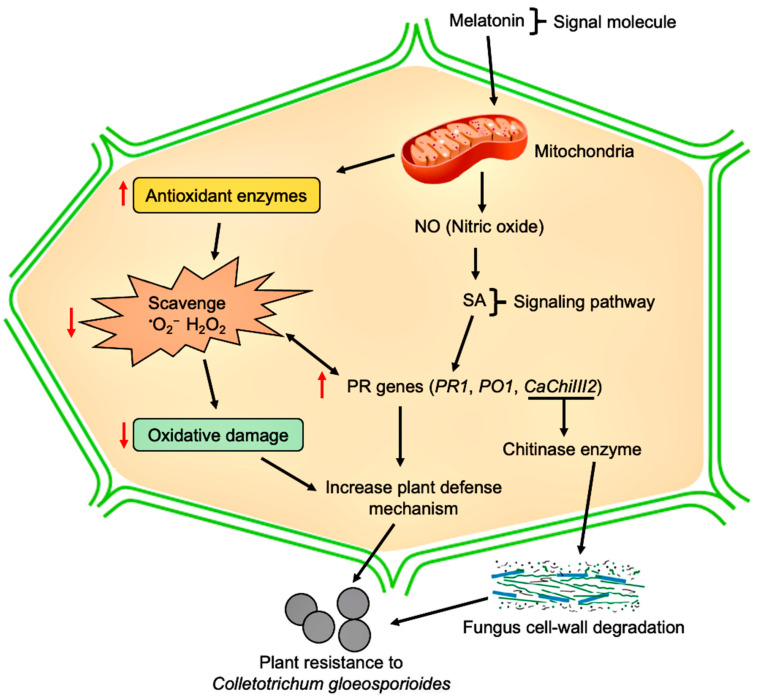
A proposed model for the mechanism of chitinase gene *CaChiIII2* in melatonin-mediated innate immunity against *C. gloeosporioides* infection.

Based on previous work and the current study, a novel approach is suggested for melatonin mediated response to *C. gloeosporioides* infection in pepper plants (Figure 10). Exogenous application of melatonin remarkably increased the transcripts level of chitinase gene *CaChiIII2* and mitigated the infection. Previous studies propose that exogenously applied melatonin works as a signaling molecule and increases the synthesis of nitric oxide (NO) and SA in the treated plants, while nitric oxide triggers the induction of SA accumulation [88,115,116]. The PR (*PR1*, *PR5*, and *CaChiIII2*) gene expression is triggered by higher amounts of SA [28,117], which in turn improves plant resistance to biotic stresses. Obviously, further work is required to explain the precise mechanisms involved in plant resistance against the fungus.

## 5. Conclusions

The present study identified the protective roles of melatonin in pepper plants. The exogenous melatonin treatment before inoculation with *C. gloeosporioides* lessened pathogen impact through the reduction in lesions and preventing its spread. This allowed plants to retain, uncouple the knockdown of *CaChiIII2*, and the expression levels of other PR genes in both control and *CaChiIII2*-silenced pepper plants activate transiently through recovering resistance. Moreover, it also kept the concentration of intracellular H_2_O_2_ at a steady-state level and increased the activities of antioxidant enzymes. In addition to inducing plant innate immunity against pathogens, melatonin application also attenuated *C. gloeosporioides* growth by inhibiting mycelia extension. Additionally, seed hydropriming with melatonin promoted the root-length and tolerance of Arabidopsis seedlings under NaCl stress. All the mentioned aspects undoubtedly explain why melatonin provides protection against anthracnose. Consequently, the exogenous melatonin treatment signifies a promising approach in guarding the pepper plant against the impact of *C. gloeosporioides* infestation. The study reveals the first work on melatonin defensive action against *C. gloeosporioides* in pepper plants. However, more investigation is required to know the mechanism of how exactly the resistance is acquired.

## Data Availability

All datasets generated for this study are included in the article/Supplementary Materials.

## Supplementary Materials

The following are available online at https://www.mdpi.com/2076-3921/10/1/7/s1, Figure S1: Virus induced gene silencing (VIGS) vector, Figure S2: Overexpression vector pVBG2307, Figure S3: Subcellular localization vector pVBG2307-GFP, Figure S4: Effect of exogenous melatonin application on the expression levels of different genes, Figure S5: Representative phenotypes of pTRV2:*CaPDS* (positive control), pTRV2:00 (negative control) and pTRV2:*CaChiIII2* (*CaChiIII2*-silenced), Table S1: The CDS sequence of chitin-binding protein gene family with accession number, Table S2: Primer pairs for qRT-PCR, Table S3: Primer pairs for knockdown of *CaChiIII2* and *CaPDS*, Table S4: Primer pairs for transformation of *CaChiIII2*, Table S5: Primer pairs for subcellular localization of *CaChiIII2*.
