# Supplementary material for: Melatonin Mitigates the Infection of Colletotrichum gloeosporioides via Modulation of the Chitinase Gene and Antioxidant Activity in Capsicum annuum L
Source: Antioxidants (Basel). 2020 Dec 23;10(1):7. doi: 10.3390/antiox10010007 (PMC7822495; doi:10.3390/antiox10010007)
Supplement: Supplementary file 1 [file antioxidants-10-00007-s001.zip › Supplementary/Supplementary Tables.pdf]

Melatonin Mitigates the Infection of *Colletotrichum gloeosporioides* via Modulation of the Chitinase Gene and Antioxidant Activity in *Capsicum annuum* L.

Muhammad Ali [1,2], Anthony Tumbeh Lamin-Samu [1], Izhar Muhammad [3], Mohamed Farghal [1], Abdul Mateen Khattak [4], Ibadullah Jan [5], Saeed ul Haq [4], Abid Khan [6], Zhen-Hui Gong [2,*] and Gang Lu [1,*]

1 Department of Horticulture, College of Agriculture and Biotechnology, Zhejiang University, Hangzhou 310058, China; alinhorti@yahoo.com (M.A.); anthonylaminsamu@yahoo.com (A.T.L.-S.); mohamedfa2016@gmail.com (M.F.)
2 College of Horticulture, Northwest A&F University, Yangling 712100, China
3 College of Agronomy, Northwest A&F University, Yangling 712100, China; izeyaar@gmail.com
4 Department of Horticulture, The University of Agriculture, Peshawar, Pakistan; mateen@aup.edu.pk (A.M.K.); saeed_ulhaq@nwsuaf.edu.cn (S.u.H.)
5 Department of Agriculture, University of Swabi, Khyber Pakhtunkhwa, Pakistan; dribad@uoswabi.edu.pk
6 Department of Horticulture, The University of Haripur, Haripur 22620, Pakistan; abidagriculturist@gmail.com
* Correspondence: zhgong@nwsuaf.edu.cn (Z.-H.G.); glu@zju.edu.cn (G.L.)

Table S1. The CDS sequence of chitin-binding protein gene family with accession number

CaChiI1 **(Capana07g001653)**
ATGAAGCTTTGTGAATTCACAGCTCTTTCTTTGTTACTTTCTCTCCTATTGCTAGCTGCATCGGCA<br>GAACAATGTGGTAAGCAGGCGGGAGGTGCACGTTGTGCCCCTGGACTCTGTTGCAGCAAATTTG<br>GCTGGTGTGGTAATACTAATGTCTATTGTGGTCCTGGAAATTGTCAAAGTCAGTGTCCTTCGGGT<br>CCCTCTCCGAAGCCACCAACCCCTGGTCCTCGTCCTGGTGGGGACATCAGTGGTGTTATCTCAA<br>ATTCTATGTTTGATCAAATGCTTAAGCATCGCAATGATAATGCATGTCAAGGAAAGGGTAATTT<br>CTACAGTTACAATGCCTTCATCAGTGCTGCCAGGTCTTTTCGTGGCTTTGGTACCACTGGTGATA<br>CCACTGCCCGAAAAAGAGAAATTGCTGCTTTCTTTGCCCAAACCTCCCATGAAACTACCGGAGG<br>ATGGCCTACAGCCCCTGATGGACCATATGCATGGGGATACTGCTTCCTTAGAGAACGAGGTAGC<br>CCCGGGGACTACTGTACACCAAGTGGTCAATGGCCTTGTGCTCCTGGAAGAAAATACTTCGGAC<br>GAGGTCCCATTCAAATCTCACACAATTACAACTATGGGCCATGTGGAAGAGCCATCGGAGTTGA<br>CCTTTTAAACAATCCTGATTTAGTTGCCACAGATCCAGTCATCTCATTCAAATCAGCTATCTGGT<br>TCTGGATGACCCCTCAATCACCAAAGCCTTCTTGCCACGATGTCATCATTGGAAGATGGCAACC<br>ATCCAGCGCTGACCGAGCAGCCAACCGCCTCCCTGGATTCGGCGTCACCACAAACATCATTAAT<br>GGTGGCTTGGAATGTGGTCATGGTAATGACAATAGGGTCCAGGATCGGATTGGGTTTATAGGA<br>GGTATTGTGGAATTCTTGGAGTTAGCCCAGGTAACAATCTTGATTGCGGCAATCAGAGATCTTTT<br>GGCAATGGACTCTTAATCGATTCTATGTAA
CaChiI2 **(Capana10g001143)**
ATGAAGCTTTGTAAATTCACAGTTCTTTCTTTACTATTTTCTCTCCTATTTCTAACTGTCTCGACA<br>GAACAATGTGGTAAGCAGGCGGGAGATGCACGTCGTGCCCCCGGACTCTGTTGCAATAAATTTG<br>GCTGGTGTGATAACACTAATGTCTATTGTGGTCCTGGAAATTGTCAAAGCCAATGTCCTTCTGGT<br>CCCACTCCGAAGCCACCTACCCTTGGCCCTCGTCCTGGTGGGGACATCAGCGGTGTAATCTCAA<br>ATTCTGTGCTTGATCAGATTCTTAAGCATCGCAATGATAATGCCTGTCGAGGAAAGGGTAATTT<br>CTACAATTACAATGCCTTCATCAATGCTGTCAGGTCTTTTTGTGGCTTTGGTACCACTCGTGATA<br>TCATTGTACGAAAAAGGGAAATTGCTCAAACCTCCCATGAAAGTACCGGATGGCCTACAGCACC<br>TGACGGACCACATGCATGGGGTTACTGCTTCCTTAGAAAACGAGGTAGCCCCGGCGACTACTGT<br>TCACCAAGTGGTCAATGGCCTTGTGCTCCTGGAAGAAAATATTTTTGA
CaChiI3 **(CA10g09850)**
ATGAGGCTAAGTGAATGGACAATTTTTTCATTACTATTTGCTCTCTTATTGCTGATGGCCTCGGC<br>AGAACAATGTGGTTCTCAGGCAGGAGGTGCACTTTGTCCCTCAGGACTCTGTTGTAGCAAATAT

GGCTGGTGTGGTGACACCGATGCCTATTGTGGTCCTGGCAATTGTCAAAGCCAGTGTCCTGGCA<br>GTCCCTCTCCCAAACCACCTACCCCTGGCCCCGGTCCTAGTGGGGACATCGGTGGTGTCATCTCA<br>AATTCCATGTTTGACCAGATGCTTAAGCATCGCAATGATAATGCTTGTCAAGGAAAGAACAATT<br>TCTACAATTACAATGCCTTCATTAATGCTGCAAGGTCTTTTCCCGTCTTTGGCACCACTGGCGAT<br>ACCACTGCCCGTAAAAGGGAAATTGCTGCTTTCTTTGCCCAAACCTCCCATGAAACTACTGGTA<br>GGTCAATTTTAATTTGA
***CaChiIII1* (Capana03g000778)**
ATGATGAGAACTAGAGAGACAGCAATTAGTCTTTTGATTCTTGCTTTGTTCCTCCTTAATGTCTC<br>TGCCTATTTATCTCTTCCCTTTCATCTTCCAACCAATAATGAAACCTTTGGCCTTGAATCATACCC<br>ACAAGGACGATGTGGAAGACAAGCTGGTGGTACAAAATGTCCTACTGGAGCATGTTGTAGTAT<br>ATCTGGTTGGTGTGGAACCACATCAGACTATTGTACTCCTAGAAAGTGTCAAAGTCAATGTGTT<br>TTTCCACCCCCACCATCATATCCAGAAGGACGATGCGGAGAACAAACTTCTTTTAAAAGATCAT<br>GTCCTTCTGGACAGTGTTGCAGTATAGCTGGTTGGTGTGGAAGCACAGGAGCCTATTGTAATCC<br>TGGTTGGTGTCAAAGTCAGTGCAAAACTATAACCTCGCCCACCAAGAATCGCATGAGAGGCATT<br>GAAAGCTTCCTGCTCAATGCTCTCTAG
***CaChiIII2* (Capana03g000780)**
ATGGAGTTTGTTGTAGTATCTACGGCTGGTGTGGAACCACATCAGACTATTGTTCCCCTGGAAG<br>ATGGTCAGAGTCAATGTCCAGACCCGTACCCAGAGGGACGATGCGGATGGCAAGCTGGTGGTA<br>AACTATGTCCTAATGGAGCGTGCTGTAGTTACAGTGGTTGGTGTGGAACAGCACGACTCTCTTG<br>TAGAACTGATTTCTGTCAAAGCCAATGTGCAACCCCGTTCCCACGGGGACGATGGGGATGGCAA<br>GCTGCTGGTAGAGGATGTCCTAATGGAGTGTGTTGTAGTGTCTCTGGTTGGTGTGGAAACACAT<br>CAGACTATTGTGCTCCCGATAAATGTCAAAAACAATGTAAAACACCATTCCCACCCCCACCTTC<br>ACCCTCACCTCCACCTCCACCTCCACCTAATTTCCCACCAGGACGATGCGGAAGGCAAGCTGGT<br>GGTAGATTATGTCCTAAGCGAAAGTGTTGTAGTAGATGGGGTTGGTGTGGAACTACGCCAGAAT<br>ATTGTGCTGATAAAAATTGTCAAAGTCAGTGCAGAAATTATCTAACCTCATCCGCCAAGAATGA<br>TATGGGAGGTCTTGGAAGCTTCTAG
***CaChiIII3* (CA03g30170)**
ATGATGAAAGCTAAAAAGACAAATGATGCATTCTCAGTTAGCTTTTTGGCTCTGGCTTTGTTCCT<br>CTTAGCAATAATTTATAAGGCCTCAGCCTACAATCTCTCTCTTCCCTCTGCTAAAAATCCTGAAG<br>GTCGACGACGACAGTGTGGTTGGGAAGGTTGCTATGAAGATTGTCCCCCTGGATCATGTTGTAA<br>CTGGGACGGTATTTGCGGAAATACAGAAGATTATTGTGGCAGATCGTTCTGTCAAAAACAATGT<br>CCACCTCCACCCCCACTTGCACCTCCACCTCCAAACCCACCTGGACGATGTGGATGGCAAGCTT<br>ATGGTACTAAGTGTCCTCCTGGATTGTGTTGTAGTACATCTGGTTGGTGTGGATCCACTGAAAAA<br>TATTGCGCCAAAGGGCGTTGTCAAAGTCAGTGCAAAAGTACTCTTACCCCTCATAATAATCCAA<br>TAATAGGTAAGCGAGAGTGTGGATACCAAGGTTGCTACAAACTATGTCCTCCTGGATCGTGCTG<br>TAGCTGGTTGGGTTGGTGCGGAACTACAGAAAAGTACTGCAGTCCAAAGAACTGTCAAAGCCA<br>ATGTCCACCTATACCCCCACCTCCACCTCCACCTCCATACGCACCTGGACGATGTGGAAGGCAA<br>GCTGATGGTAGAAAATGTCCTCCTGGATTGTGCTGTAGTGCATCTGGTTGGTGCGGAACCACAA<br>AATTCTATTGCGCCAAAGAGTGGTGTCAAAGTCAGTGCAAGAGTACTACTCTTGCCTCGTCTATT<br>ATTAAGAGCTTGTTGCTCGACGGTACGGTTTCTAGCTAG
***CaChiIII4* (CA03g30180)**
ATGCAAGCTGGTGGAACAAAATGTCCTCCAGGATTGTGTTGTAGTTTATCTGGTTGGTGCGGAG<br>CCACAGAAGAGTATTGCCGCGAAGGGTGGTGTCAAAGCCAGTGCAAGAGTAATCTTACCCCTC<br>ATAATAATTCACTAATGGCTAAACGAGAGTGCGGATACCAAGGTTGCTATAAAAAATGTCCTCC<br>TGGATCGTGTTGTAGCAGGTGGGGTTGGTGCGGAACTACAGAAGAGTTTTGCACTAAATACTTC<br>TGTCAAGATCAATGTGCGCCTCCACCTGCACCTCCACCTCCACCTCCATACGCACCTGGAAGAT<br>GTGGAATGCAAGCTGATGGTAGAAAATGTCCTCCTGGATTGTGTTGTAGTTCATCTGGTTGGTG<br>CGGAACCACAAAACACTATTGCGCCAAAGAGTGGTGTCAAAGTCAGAGCAAGAGTACTACTCT<br>TACCTCGTTTGTTATTGAAAGCTTGTTGCTCAATGGTACGGTGTCTAGCTAG
***CaChiIII5* (CA03g30190)**
ATGAGAGAGATTATAATTAGCCTTTTAGCTCTGGCATTGTTCCTCCTTAAGGTCTCAGCCAAGCT<br>ATCTGATGTTCCCTTTTATTTGCCAGCCAATGAAACATTTGGCCTTGAACTAATAAGGGAACGTA<br>ATACGTCAGACCTAGCCCAGTCATTATTAGCACGGAGTCGATGCGGATGGCCAGCTGGCGGTAG<br>ATGGTGTCCAGAAGGACAGTGTTGTAATTTTGATGGTTGGTGTGGAACCACAAGTGCCTATTGT

GGTGAAAATATGTGTGATTTCCAGTGTCCAGGTCCGATCCGAGTGAGACGATGCGGAATGCAAG<br>CTGGTGGTCGTCCATGTCCTACTGGACAATGTTGTCGGGACACAGTCTCTCTGGTTGGTGTGGAA<br>CCACTTCAATCTATTGTTCTCGTGAAGAATGTCAAAGTCAGTGAGAAAGGCCACCACCACCCCC<br>GTCTCCACCACCCCCACCCCCACCATTCCCACAGGGACGATGCGGAAAGCAAGCTGCTGGTAGA<br>GCATGTCCTACTGGAGTGTGCTGTAGTATATGGGGCTGGTGTGGAACCACACGTAACTATTGTG<br>GTTCTCCCTACTGTCAAAGTCAGTGCAAGGGCGGTCTAA
CaChiIII6 **(Capana07g001180)**
ATGAGAGGAAGCAGTGCATTAGTTAGCCTTTTATCTCTGGTTTTGTTGTTCCTCCTTGAGGTCTC<br>GGCCAATCCATCTCATCCCATTCATTTGCCGGCCAATGAAACGATCCTTGGCCAGCTTGAACTA<br>GCCGAGGGGGAGGGACAATGCGGAAAACAAGGTGATGGTAAAGAATGTCCTAGTGAAGAGTG<br>TTGTAGTTACTGGGGTTGGTATGGAACCGGACCAGCTTACTGTGCTTCTGAAACCTGTCAAAGT<br>CAATGCCAAGAGCCAGAAGAGCCAGGTTTATGCGGAATACAAGGTGGTGGTAAAAAATGTCCT<br>AGTGGACAGTGTTGTACTGTATTTGGTTTGTGTACAGCAGCATCAGATATGTGTGAGCATTTTTG<br>TCAAAGTCAGTGCCCAACTACTCTAAAGAAGAACAGCATGAGAGGCATTCAAAGCTTCTTCATC<br>AATGCTCTCTAA
CaChiIII7 **(Capana07g001181)**
ATGAAAGAAACTGCATTAATTAGCGTTTTAGCTCTAGCTTTATTCCTCCTTAAGGTCTCAGCCAA<br>AACATCTCTTCCCTTTCATTTGTCAGACAGTTCAGCATCATCATCATCAGCCAAGCTATCAGGCT<br>TACCGACCCTATGCGGAGAAGCTGTTGGTGGTAGAGAATGCCCTGATGGGAAGTGTTGTAGTCT<br>AGAGGGTTATTGTGGAACAGGAGAAGCCTACTGTGCTCCTGAAAACTGTCAAAGTAATTGCGAT<br>GAGCCACCAGAGCCAGAGCTAGAATGCGGAGATCAAGCTGGTGGTAAAGAATGTCCTAATGGA<br>GAGTGTTGTAGTATATTTGGTTCGTGTGGAACCACAGAAGACCATTGTTGGAAACCTTTTTGTCA<br>AAGTCAGTGCAATGAGCCACCATTGCCACCAGAGCCAGAGAGATGCGGAAGGGAAGGTGGTGG<br>TAAAGAATGTCCTACGGGAGAGTGTTGTAGTGTATTTGGTTCCTGTGGAACCACAGAAGAACAT<br>TGTGAGGAACCTTTTTGTCAGAGTCAGTGCAAAACTAGTCTAAACAAGAATCGCATGCTGAGAG<br>GCACTCGAAGCTTCTTCCTCAATGCTCTCTAA
CaChiIV1 **(CA00g54030)**
ATGAACTTCTCTTCATCAACAAAATATTTCTTTCTCTTTGTAGCATTAGCTATAATAGCTGATGT<br>ACCAAGACTAATCTTGGCACAAAACTGTGGGTGTGCAGCAAATTTATGTTGTAGCAAATGGGGT<br>TATTGTGGAGAGGGAAAGGATTATTGTGGTGAAGGGTGTCAAGGGGGGCCATGTTTTAGTACTA<br>CACCATCAGGCAATAATGGCGGTTCAGTTTCTGATATTGTATCTGATGCATTCTTTAATGGGATA<br>GCTGATCAAGCCGCTTCTAATTGTGAAGGAAAAGGGTTTATTCAAGGGATAAATTCTTTGAAG<br>CTCTTAAATCTTATCCTAACTTTGGAACTGTGGGTTCTAATGATGACTCTAAACGTGAAATTGCT<br>GCTTTCTTTGCTCATGTCACCCACGAAACTGGCCACATGTGCTACATAAATGAGATAAATGGTC<br>CATCAGGCGACTATTGTGATGAGGACAACAAAGAGTACCCTTGTGTATCAGGCAAGAACTACTA<br>TGGTCGAGGACCAATTCAACTATCATGGAACTTCAACTACGGACCTGCTGGAAAATCCATTGGA<br>TTTGATGGCCTAAATGACCCTGACATAGTTGCAAGAGATGCTGTTATTTCCTTCAAGACAGCATT<br>GTGGTATTGGATGAACAATTGTCATTCACTAATTACTTCTGGACAAGGTTTTGGTCCAACTATTA<br>GAGCTATTAATGGTAGACTTGAATGTGATGGTGGTAATCCTCAAACTGTTGCTAGAAGGGTTGA<br>GTATTACACTCAGTATTGTCAACAACTTGGTGTTGATGCTGGGGATAATCTCACGTGTTAG
CaChiIV2 **(Capana06g002084)**
ATGTTGGCTCAGAATTGTGAGTGTGAAGAAGGATTGTGTTGTAGTAAATGGGGTTATTGTGGCA<br>TTGGAAATGGTTATTGTGGAAAAGGCTGCCAAGGAGGGCCATGTTATTTTAATATTTCACCAAA<br>ATATAATGTTAAGAGAGTTGCTGAAATTGTTACTGAATCATTCTTTAACGGGATTTCTGATCAAG<br>CTGATTCAAATTGTGAAGGCAAAGGTTTTTTCACAAGATCTGTGTTTCTTGAAGCTGTCAAGTGC<br>TATCCTGAATTCGGGACTGTTGGTTCTTCTGATGATAATAAGCGCGAAATTGCTGCTTTCTTTGC<br>TCATGTCACCCATGAGACTGGACAAATGTGCTACATAAATGAGATAAATGGTGCATCTAGGGAC<br>TATTGTGATATGACAAACTATTTGTACCCTTGTGTCTCTGGAAAGAATTATTACGGCCGAGGACC<br>GATCCAACTATCATGGAACTTCAATTATGGACCAGCCGGAAAAGCCATCGGATTTGATGGCCTA<br>AATGATCCCGATATCGTAGCTAGAGATAGTCTTATATCATTCAAGACAGCCTTGTGGTACTGGA<br>TGAATAATTGCCATTCTCTATTAACTTCTAGACAAGGTTTCGGAGCGACAATTCGAACCATTAAT<br>GGCCTTCTTGAGTGTAATGATGCCAATCCTGAGGCTGTTGCCAGAAGGGTTCAATATTACATCG<br>AGTATTGCGAGCAACTTGGTGTAGACCCTGGGGATAATCTCTCTTGTTAG
CaChiVI1 **(CA07g09480)**

ATGAAGTTTCAGGTGGTAATTTTGGTGCTTTTGGCCTTGTTGTTAACCACAACAAGTGCTCAACA ATGCGGAAGACAAGCTGGAGGGCGTGCCTGTGCAAACAGGTTGTGCTGCAGCCAATACGGGTT CTGTGGTACGACTCGGGCATACTGTGGAGTTGGTTGCCAGAGTAATTGTGGCCGTTATGCCACT GACACCACTGGCGAAGCTGAAAATGTTGACAATGATGAACACAAGAATAATGACGGTCCCAAC TAA
***CaChiVI2* (Capana08g001237)**
ATGGAGAAGCTAAGTACTACTGCTCTTTTGCTGTCTTTGGTCCTCTTCATCATAGCCGCAGTTGC AAACGCACAACAGTGTGGGAGGCAAAGGGGCGGAGCGGTATGCAGCGGGAGCTTGTGTTGCAG CCAGTATGGTTGGTGTGGATCGACACCCGAATACTGTTCACCTAGCCAAGGTTGTCAGAGCCAA TGCGGTGGCAGTGTACCAACTCCAACTCCAGGAGGAGGTGGGGCTAGCGCGCAAAATGTACGT GCAACATATCATTTGTATAACCCGCAGAATGTTGGGTGGGACTTGAATGCGGTTAGTGCTTATT GCTCTACTTGGGATGCTAATAAGCCTTTGGCCTGGAGGAGCAAGTATGGTTGGACTGCTTTCTGT GGTCCTGTTGGACCTCGTGGTCGAGACTCATGCGGCAAGTGCTTAAGGGTGACAAACACACGCA CAGGAGCTCAGACGATAGTGAGAATCGTGGATCAATGCAGCAATGGTGGACTAGATTTAGACG TTAACGTTTTCCGTCAAATCGATACGGACGGAGTAGGAAATCAACGAGGCCACCTTATTGTGAA CTACCAGTTTGTTGATTGTGGTGATAACTGA
***CaChiVI3* (CA08g10220)**
ATGGAGAAACGAACTAGTACTACTATTCTTCTAGCCCTAGTCCTCTTCATCGTATCCGGAGTTGC CAACGCACAGCAGTGCGGAAGGCAAAGGGGCGGAGCCTTATGCGGCGGAAACTTATGTTGCAG CCAATTTGGATGGTGTGGATCGACACCCGAATACTGTTCACCTAGCCAAGGTTGCCAGAGCCAA TGCAGTGGAAGTGGTCCGACTCCAGAAGGAAGCGCGCAAAATGTACGTGCAACGTATCATTTGT ATAACCCGCAGAATGTTGGTTGGGACTTGAATGCTGTTAGTGCTTATTGCTCGACATGGGATGC TAATAAGCCTTTGGCTTGGAGGAGTAAGTATGGTTGGACTGCTTTCTGTGGTCCTGTTGGACCTC GTGGTCAAGCCTCCTGTGGCAAGTGCTTAAGGGTCACAAACAGACGAACCAGAGCTCAAACAA CGGTGAGAATCGTGGATCAATGCAGCAACGGTGGACTAGACTTGGACATTAACGTTTTCCGGCA AATCGACACAGACGGAGTGGGAAATCAACAAGGTCACCTTATGGTGGACTACCAATTTGTTAAT TGCGGTGACAATGTGAATGTTCCTCTCTTGTCTGTAGTTGACACACAATGA
***CaChiVI4* (CA12g08860)**
ATGTCATATATAAGCAATTACCCTATATTCCATAGAGAGAAAAAAGTAATAACAAAAACGATG AGATTTTCATTAGCTCTTGTCTTTGTGATTTTGGCCCTGTTGCTAACTACTACTTATGCAGAACAA TGTGGTAGACAAAACCATAAGCGTAAGTGTCCCAACAAGCTGTGTTGCAGCAAGTTTGGATGGT GTGGCACTAGTTGTGACTACTGTGGAGCTGGCTGCCAGAGAAACTGTAACAAAGGTTGCGCCAC TACCATGTTCGCCAATGAAACCGTCAACAATAGTGGCGAACACTTGGATGGTGGTAACTTAAAC TGA

Table S2. Primer pairs for qRT-PCR

Gene name	Primer sequence (5'→3')	Product length/AN
CaChiI1	F: AAACCTCCCATGAAACTACCG<br>R: GTTTAAAAGGTCAACTCCGATGG	227
CaChiI2	F: GTTTAAAAGGTCAACTCCGATGG<br>R: CAGCATTGATGAAGGCATTGT	116
CaChiI3	F: CTATTTGCTCTCTTATTGCTGATGG<br>R: CAGGACCACAATAGGCATCG	133
CaChiIII1	F: TCTTCCCTTTCATCTTCCAACC<br>R: AGTTTGTTCTCCGCATCGTC	229
CaChiIII2	F: CTCCACCTCCACCTAATTTCC<br>R: CCACACCAACCCCATCTAC	100
CaChiIII3	F: TGGTGCGGAACTACAGAAAAG<br>R: TTCTACCATCAGCTTGCCTTC	127
CaChiIII4	F: GGAATGCAAGCTGATGGTAGA	110

	R: CTCTGACTTTGACACCACTCTT	
CaChiIII5	F: GAAAGCAAGCTGCTGGTAGA<br>R: TGACTTTGACAGTAGGGAGAAC	106
CaChiIII6	F: GTCCTAGTGAAGAGTGTTGTAGTT<br>R: CGCATAAACCTGGCTCTTCT	114
CaChiIII7	F: TCAGCCAAAACATCTCTTCCC<br>R: CATCAGGGCATTCTCTACCAC	121
CaChiIV1	F: TCTTTGCTCATGTCACCCAC<br>R: ATCTCTTGCAACTATGTCAGGG	227
CaChiIV2	F: TCAAGTGCTATCCTGAATTCGG<br>R: CTTTCCAGAGACACAAGGGTAC	182
CaChiVI1	F: CAATACGGGTTCTGTGGTACG<br>R: CAACATTTTCAGCTTCGCCAG	103
CaChiVI2	F: TGGGACTTGAATGCGGTTAG<br>R: TCACTATCGTCTGAGCTCCTG	178
CaChiVI3	F: CGACATGGGATGCTAATAAGCC<br>R: CGTTGTTTGAGCTCTGGTTCG	143
CaChiVI4	F: GTCTTTGTGATTTTGGCCCTG<br>R: TTGGCGAACATGGTAGTGG	191
CaPR1	F: TGGAGACTGCAGGATGCAACACT<br>R: TACCACCCATTGTTGCACCGAAC	AF053343.2
CaPO1	F: CCCTTCAATCGACGCATCCTTTC<br>R: CCAAGAAATCCCCTGAGCCCTA	AF442386.1
CaDEF1	F: CAAGGGAGTATGTGCTAGTGAGAC<br>R: TGCACAGCACTATCATTGCATAC	AF442388.1
CaPO2	F: TGATTGCTTTGTTCAGGGTT<br>R: ATGATGGACCTCCAACGAGA	DQ489711.1
CaAP2/ERF064	F: CAACTCCTTCTTCTTGCTCTTC<br>R: CGCCTCCTAACACCTCGGTA	XM_016712649.1
CaBPR1	F: CTGGTGCCGTGAAGATGTGGGT<br>R: TACCACCCATTGTTGCACCGAA	AF053343
CaASMT1	F: GAGACACCCTCTATGGACTTAC<br>R: TGAGAATTGGATTGTTTTGG	CA09g13730
CaSNAT1	F: GAGGGTAATGCAGAAAAGAA<br>R: ATGGGAAGGATCAACAAGAA	CA10g10570
CaSNAT2	F: TTGGGATGTGGTTGTGG<br>R: CGGGGCTCCGAATAAA	CA11g02340
CaMPK3	F: GAAGGGAGTTTTCTGATGTTT<br>R: ATGTATTTTAGCCCACGGA	CA06g06270
CaMPK6	F: ATCCCAGAAGGAGAATAACAG<br>R: CAAGAAAACACCTGGACACT	CA03g14570
CaCAT1	F: GTTGGTCGCTTGGTATTGA<br>R: TTGGGAGCGTTAGGTGG	CA04g22770
CaAPX1	F: CAAGGAGCGTTCTGGTTT<br>R: CTGATGGCAACTGTAAAAGCCCTTC	CA06g06240
CaICS1	F: TGCTTTTGGCTCATTTTATT<br>R: TCATCTATTGCCCTCTGGTAT	CA06g19210

F: Forward primer R: Reverse primer

Table S3. Primer pairs for knockdown of *CaChiIII2* and *CaPDS*

Primer Name	Primer sequence (5'→3')	Enzymes
CaChiIII2	F: CCGGAATTCAGATGGTCAGAGTCAATGTCCA	*EcoR1*
	R: CCGCTCGAGCAGAGACACTACAACACACTCC	*Xho1*
CaPDS	F: GGGGAATTCTGTTGTCAAAACTCCAAGGTCTGTA	*EcoRI*
	R: GGGGGATCCTTTCTCCCACTTGGTTCACTCTTGT	*BamH1*

Table S4. Primer pairs for transformation of *CaChiIII2*

Primer Name	Primer sequence (5'→3')	Enzymes
CaChiIII2	F: GCTCTAGACCTAATGGAGTTTGTTGTAGTATC	*Xba1*
	R: GGGGTACCGAAGCTTCCAAGACCTCC	*Kpn1*

Table S5. Primer pairs for subcellular localization of *CaChiIII2*

Primer Name	Primer sequence (5'→3')	Enzymes
CaChiIII2	F: GCTCTAGACCTAATGGAGTTTGTTGTAGTATC	*Xba1*
	R: GGGGTACCGAAGCTTCCAAGACCTCC	*Kpn1*
