# Supplementary material for: Melatonin Mitigates the Infection of Colletotrichum gloeosporioides via Modulation of the Chitinase Gene and Antioxidant Activity in Capsicum annuum L
Source: Antioxidants (Basel). 2020 Dec 23;10(1):7. doi: 10.3390/antiox10010007 (PMC7822495; doi:10.3390/antiox10010007)
Supplement: Supplementary file 1 [file antioxidants-10-00007-s001.zip › Supplementary/Supplementary Figures.pdf]

Melatonin Mitigates the Infection of *Colletotrichum gloeosporioides* via Modulation of the Chitinase Gene and Antioxidant Activity in *Capsicum annuum* L.

Muhammad Ali [1,2], Anthony Tumbeh Lamin-Samu [1], Izhar Muhammad [3], Mohamed Farghal [1], Abdul Mateen Khattak [4], Ibadullah Jan [5], Saeed ul Haq [4], Abid Khan [6], Zhen-Hui Gong [2,*] and Gang Lu [1,*]

[1] Department of Horticulture, College of Agriculture and Biotechnology, Zhejiang University, Hangzhou 310058, China; alinhorti@yahoo.com (M.A.); anthonylaminsamu@yahoo.com (A.T.L.-S.); mohamedfa2016@gmail.com (M.F.)
[2] College of Horticulture, Northwest A&F University, Yangling 712100, China
[3] College of Agronomy, Northwest A&F University, Yangling 712100, China; izeyaar@gmail.com
[4] Department of Horticulture, The University of Agriculture, Peshawar, Pakistan; mateen@aup.edu.pk (A.M.K.); saeed_ulhaq@nwsuaf.edu.cn (S.u.H.)
[5] Department of Agriculture, University of Swabi, Khyber Pakhtunkhwa, Pakistan; dribad@uoswabi.edu.pk
[6] Department of Horticulture, The University of Haripur, Haripur 22620, Pakistan; abidagriculturist@gmail.com
* Correspondence: zhgong@nwsuaf.edu.cn (Z.-H.G.); glu@zju.edu.cn (G.L.)

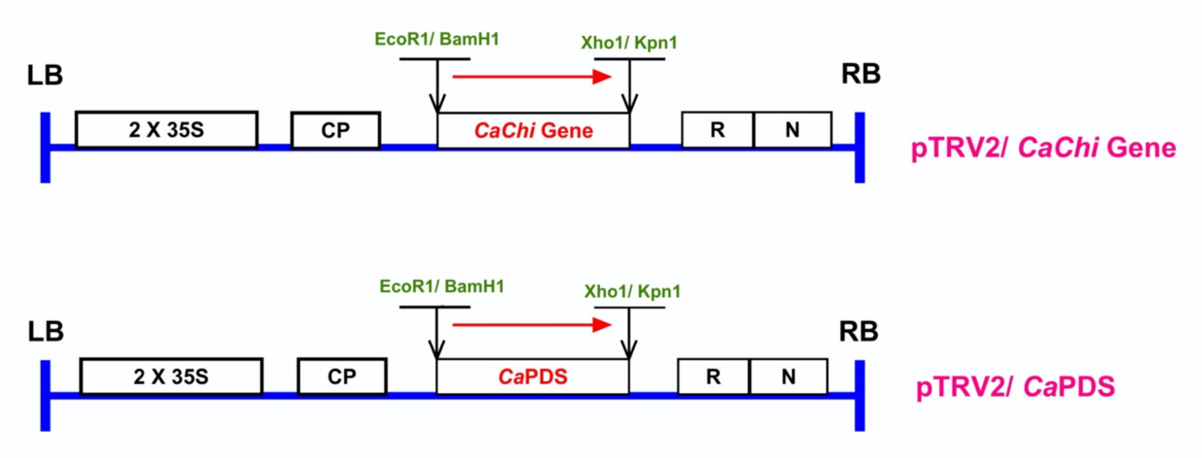

Figure S1. Virus induced gene silencing (VIGS) vector.

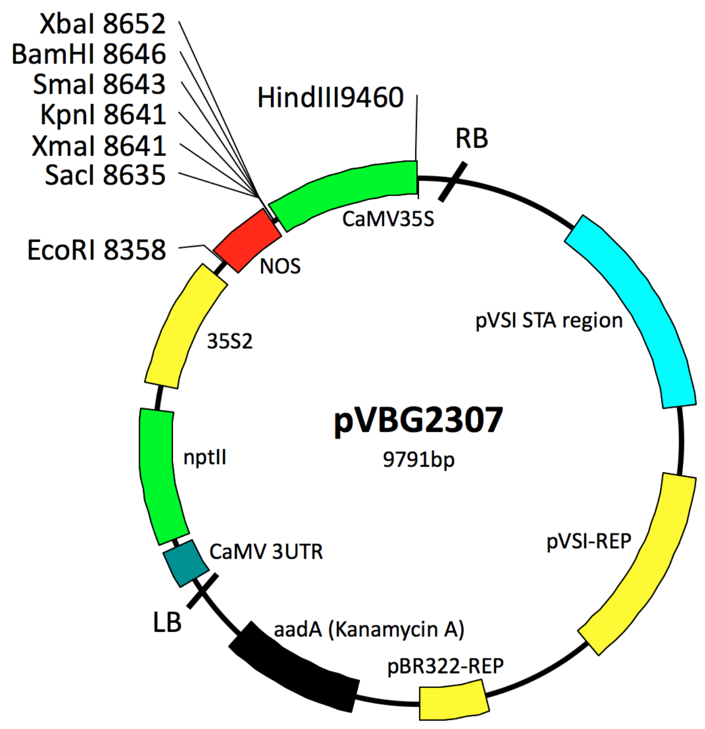

Figure S2. Overexpression vector pVBG2307.

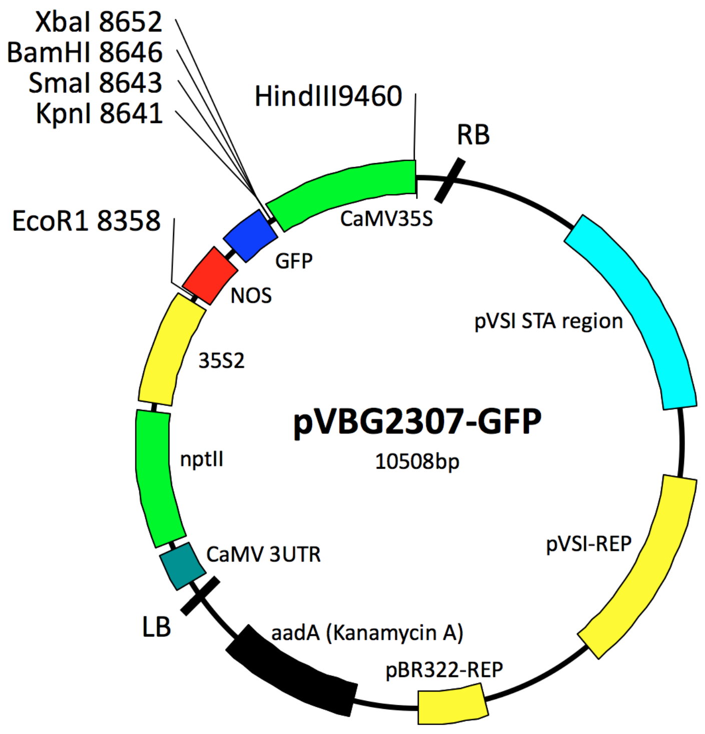

Figure S3. Subcellular localization vector pVBG2307-GFP

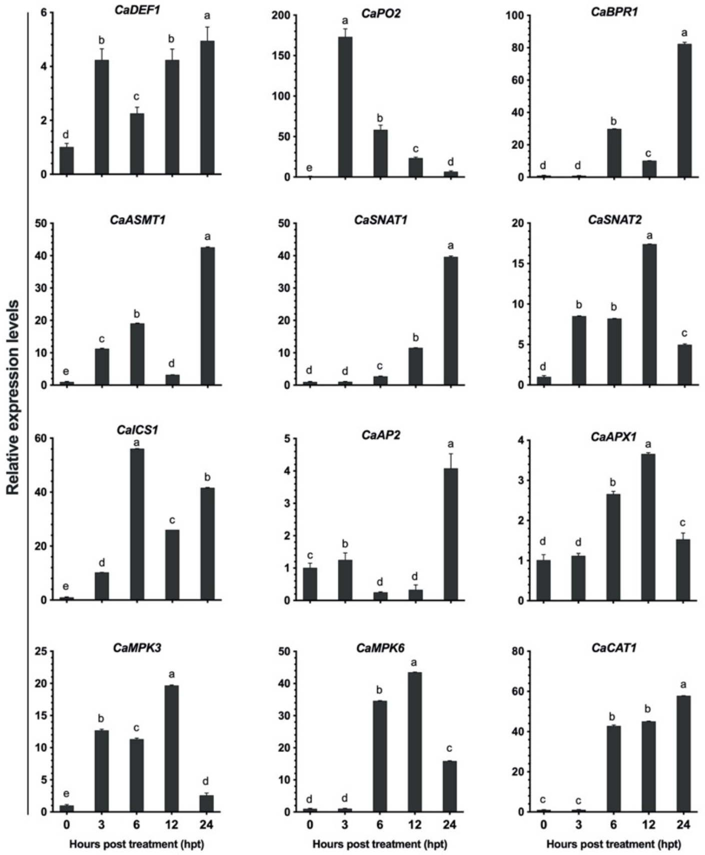

Figure S4. Effect of exogenous melatonin application on the expression levels of different genes. Tissues samples were taken at different intervals (0, 3, 6, 12, and 24-hours post-treatment) and were observed through qRT-PCR. The mean values ± SDs of 3 replications are given. Lower case letters (a–f) on each histogram show the respective differences at $P \leq 0.05$ using the DMR test.

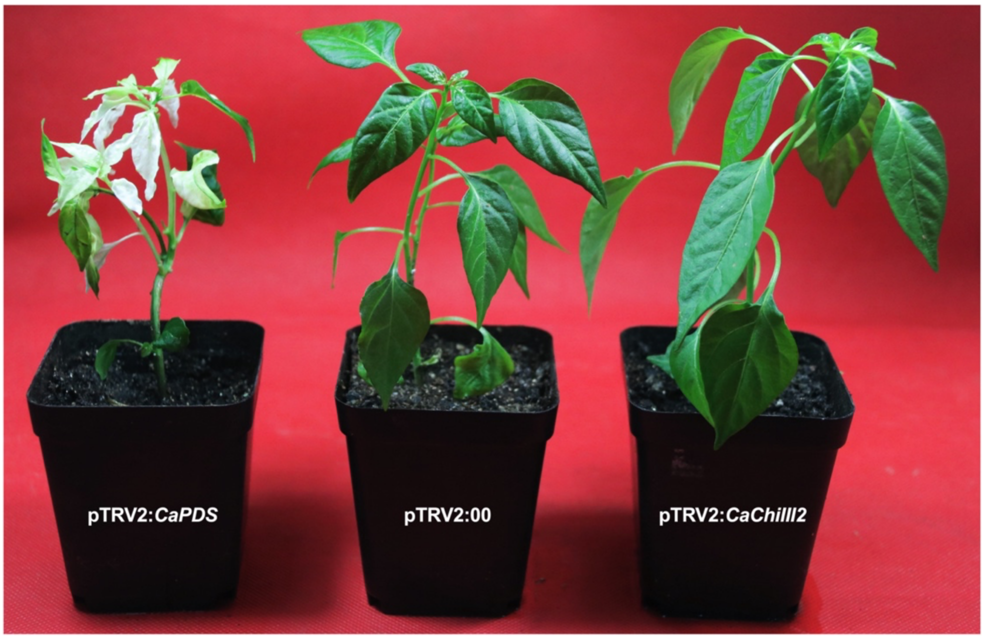

Figure S5. Representative phenotypes of pTRV2:*CaPDS* (positive control), pTRV2:00 (negative control) and pTRV2:*CaChiIII2* (*CaChiIII2*-silenced)
